# Functional specialization of parallel distributed networks revealed by analysis of trial-to-trial variation in processing demands

**DOI:** 10.1152/jn.00211.2022

**Published:** 2022-10-05

**Authors:** Lauren M. DiNicola, Oluwatobi I. Ariyo, Randy L. Buckner

**Affiliations:** ^1^Department of Psychology, Center for Brain Science, Harvard University, Cambridge, Massachusetts; ^2^Athinoula A. Martinos Center for Biomedical Imaging, Massachusetts General Hospital, Charlestown, Massachusetts; ^3^Department of Psychiatry, Massachusetts General Hospital, Charlestown, Massachusetts

**Keywords:** association cortex, frontoparietal control network, cognitive control, hippocampus

## Abstract

Multiple large-scale networks populate human association cortex. Here, we explored the functional properties of these networks by exploiting trial-to-trial variation in component-processing demands. In two behavioral studies (*n* = 136 and *n* = 238), participants quantified strategies used to solve individual task trials that spanned remembering, imagining future scenarios, and various control trials. These trials were also all scanned in an independent sample of functional MRI participants (*n* = 10), each with sufficient data to precisely define within-individual networks. Stable latent factors varied across trials and correlated with trial-level functional responses selectively across networks. One network linked to parahippocampal cortex, labeled Default Network A (DN-A), tracked scene construction, including for control trials that possessed minimal episodic memory demands. To the degree, a trial encouraged participants to construct a mental scene with imagery and awareness about spatial locations of objects or places, the response in DN-A increased. The juxtaposed Default Network B (DN-B) showed no such response but varied in relation to social processing demands. Another adjacent network, labeled Frontoparietal Network B (FPN-B), robustly correlated with trial difficulty. These results support that DN-A and DN-B are specialized networks differentially supporting information processing within spatial and social domains. Both networks are dissociable from a closely juxtaposed domain-general control network that tracks cognitive effort.

**NEW & NOTEWORTHY** Tasks shown to differentially recruit parallel association networks are multifaceted, leaving open questions about network processes. Here, examining trial-to-trial network response properties in relation to trial traits reveals new insights into network functions. In particular, processes linked to scene construction selectively recruit a distributed network with links to parahippocampal and retrosplenial cortices, including during trials designed not to rely on the personal past. Adjacent networks show distinct patterns, providing novel evidence of functional specialization.

## Graphical abstract

**Figure:**
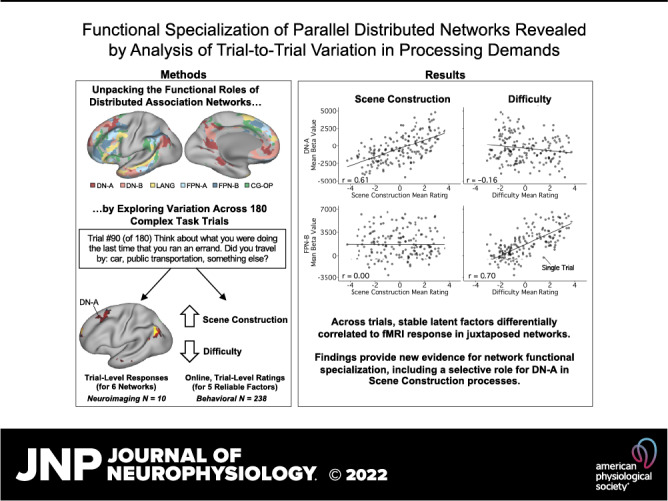

## INTRODUCTION

Diverse higher-order functions, including autobiographical memory, spatial navigation, and social inference, have been attributed to a large monolithic network known as the default network (DN; 1–3; see also Refs. 4–7). This network extends into rostral temporal and prefrontal association cortex leading to its description as the apex higher-order association network (8, 9). Considerable attention has been given in recent years to understand the processing contributions of the DN to human cognition (e.g., Refs. 10–16).

One challenge for understanding processes supported by the DN is that most prior studies rely on group-averaged data, which necessarily blurs anatomical details (see Refs. 17–19). Recent explorations within intensively scanned individuals reveal that the DN comprises at least two distinct parallel networks (9, 20; see also Ref. 21). These networks,termed DN-A^1^ and DN-B, contain features of previously hypothesized DN subsystems (e.g., 23, 24) but are fully distinct (20), raising questions about functional differentiation. Both networks possess regions distributed across multiple association zones with side-by-side juxtapositions throughout the cortex, sometimes on opposite sides of the same sulcus (25). Given these spatial arrangements, unraveling their distinct processing contributions has been hampered by spatial averaging over individuals.

Supporting functional heterogeneity, within-individual analyses suggest that DN-A is preferentially recruited by tasks targeting episodic remembering and imagining the future, and DN-B by tasks targeting social inferences. Contrasting these task domains reveals a replicable double dissociation (26; see also Refs. 27–29). Adding further evidence for domain specialization, separate regions of DN-A and DN-B within the posterior midline differentially respond to spatial versus social content (30, 31; see also Refs. 21, 32).

However, there is a second complicating factor for understanding the processing contributions of these juxtaposed networks. The tasks that elicit activation responses in these networks are often complex, involving temporally extended trial structures that encourage rich and varied mental constructions (e.g., Refs. 33, 34). In this sense, much like the spatial blurring that has led to ambiguities in the existing literature, the mixing of multiple task components in the lengthy task trials that activate the DN also leads to ambiguities. Open questions thus remain about the nature of the underlying processes that these recently identified, parallel networks support, as well as those that differentiate DN-A and DN-B from other juxtaposed networks. Answering a question about one’s past, for example, involves multiple processes traditionally associated with episodic memory retrieval (e.g., Ref. 35), as well as component processes that might generalize beyond episodic memory, such as constructing a scene in a spatially coherent context (e.g., Refs. 5, 33) and domain-general controlled processing (36–39; see also Refs. 40, 41).

**Figure 1.**
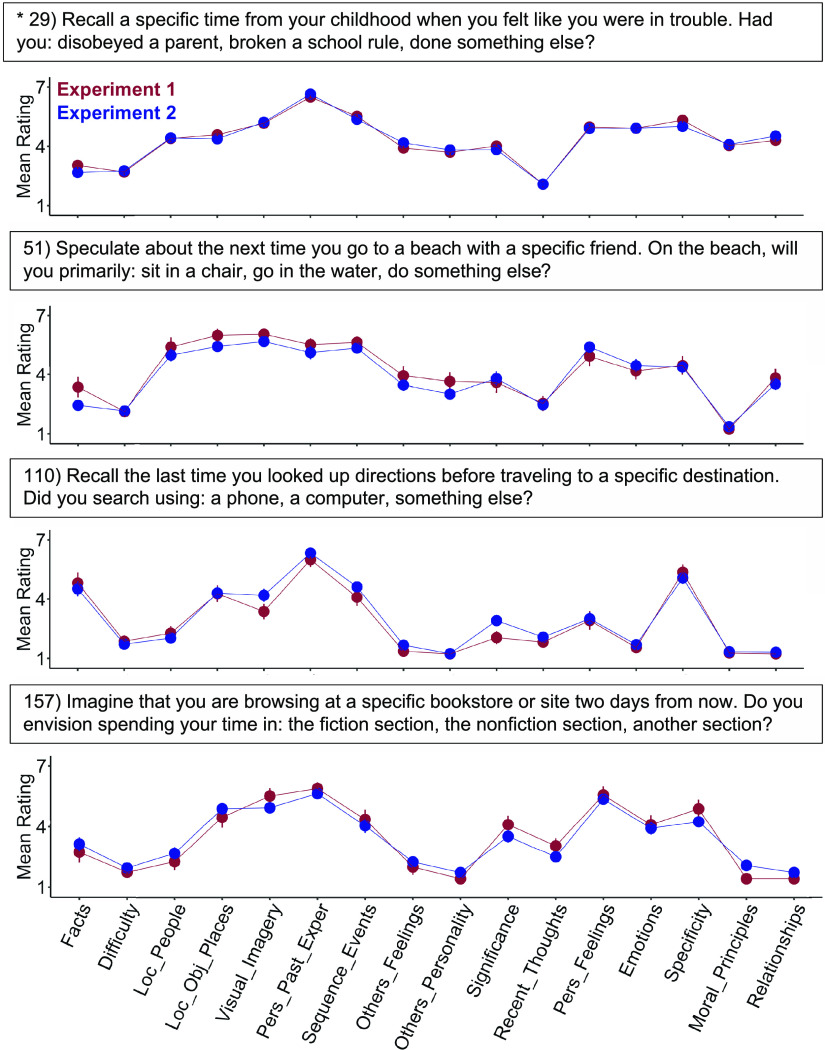
Behavioral ratings illustrate unique and reliable strategy use patterns. Mean strategy ratings from independent groups of behavioral participants show striking similarity (red: *exp 1*, blue: *exp 2*). Four example trials are displayed, chosen from the original “target” conditions designed to demand episodic projection (e.g., remembering and imagining the future). Above each plot is the actual question the participants viewed; below is the measured strategy pattern. The strategies plotted on the *x*-axis are listed in Table 1. The trials share high ratings for strategies relevant to episodic memory, as intended, such as consideration of the personal past (Personal_Past_Exper), events (Sequence_Events), and mental scenes (Visual_Imagery, Loc_Obj_Places). High intertrial variability on other strategy dimensions highlights the exploratory opportunity (see also Fig. 2). For example, Difficulty was low for some trials and higher for others. Each point shows a mean strategy rating across participants with standard error bars. Sample sizes for estimating each trial varied, always with *N* > 16. In the present four example trials, *N* = 136 (*exp 1*) and *N* = 238 (*exp 2*) for trial 29; *N* = 17 and *N* = 39 for trial 51; *N* = 22 and *N* = 42 for trial 110, and *N* = 19 and *N* = 40 for trial 157. Exper, Experiences; Pers, Personal. *A repeated trial with a larger sample size.

**Figure 2.**
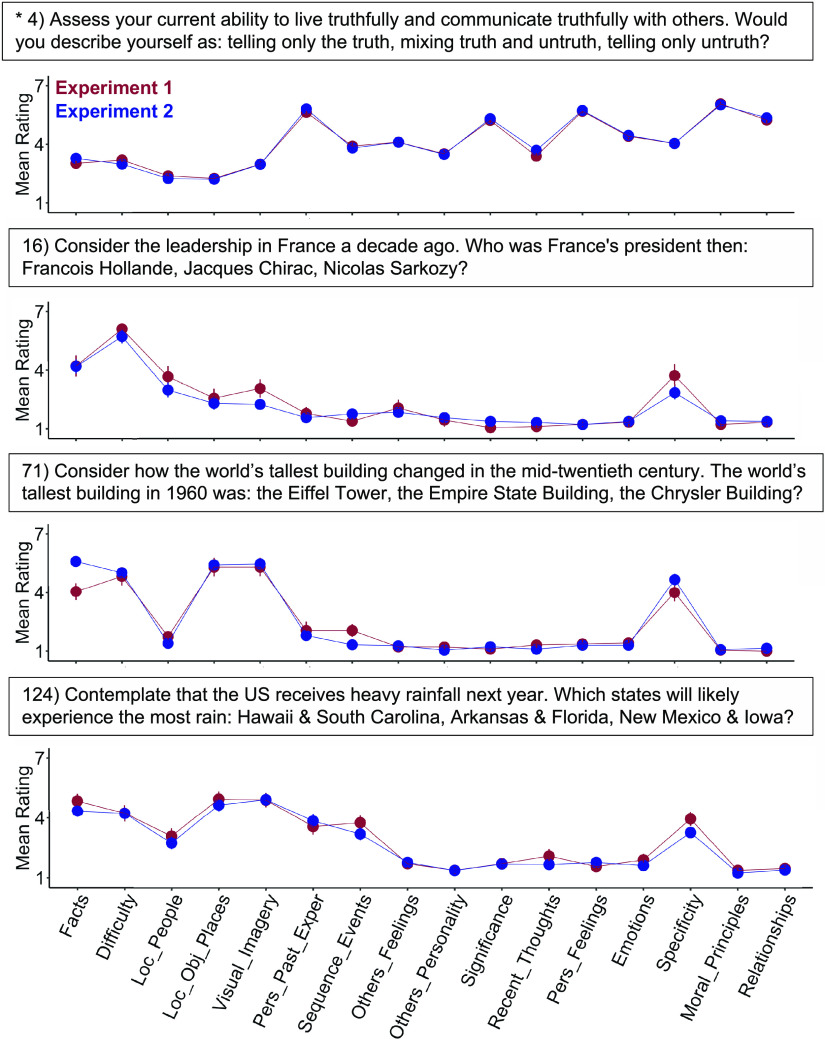
Strategy use patterns differ markedly among the control trials. Strategy patterns are shown for four trials taken from the control conditions. For each trial, mean strategy ratings from independent groups again reveal notable overlap (red: *exp 1*, blue: *exp 2*). The four trials were selected from the original “control” conditions, designed to minimize demands on episodic projection. Most of these trials show lower reliance on the personal past (Pers_Past_Exper) and greater use of facts than the target trials in Fig. 1. The control trials also reveal marked variability. Multiple trials involve strategies related to mental scenes (Visual_Imagery, Loc_Obj_Places), for example, or to considering unfolding events (Sequence_Events). Each point shows a mean strategy rating across participants with standard error bars. As in Fig. 1, sample sizes for estimating each trial varied. In the present four example trials, *N* = 117 (*exp 1*) and *N* = 238 (*exp 2*) for trial 4; *N* = 18 and *N* = 37 for trial 16; *N* = 19 and *N* = 40 for trial 71, and *N* = 21 and *N* = 40 for trial 124. Exper, Experiences; Pers, Personal. *A repeated trial with a larger sample size.

Here, we explored network functions using a behavioral approach to probe trial-to-trial variation in processing demands across a diverse set of previously scanned task trials targeting episodic remembering and imagining the future. The approach did not assume specific relations between component processes and individual network responses but allowed relations to emerge to the degree that trial-to-trial variation in processing demands selectively associated with network responses. For each trial, participants were asked questions that encouraged them to remember or imagine distinct scenarios (see examples in Figs. 1 and 2). The questions were designed to vary in self-relevance (self or non-self) and temporal orientation (past, present, or future), and afforded considerable opportunity to adopt varied strategies. Prior analyses of these functional MRI data focused on predetermined contrasts between conditions that grouped many trials together (26). Plotting network responses across separate trials within each condition revealed large signal variations, well beyond that expected by measurement error, reinforcing that there is unaccounted for trial-level variation and creating a novel experimental opportunity.

Specifically, we behaviorally assessed how people responded to each individual trial question, to quantify trial-level properties for comparison to network activity (as indirectly measured by the functional MRI signal). Evidence from prior studies supports that assessing how people respond to complex stimuli can provide insight into processing demands (e.g., related to memory encoding – 42; emotion discrimination – 43; and differentiating physical from emotional pain – 44). Most directly relevant to the present study, Andrews-Hanna et al. (23) collected strategy ratings from both scanned participants and an independent behavioral group and found that composites of strategy ratings tracked activity in network regions of interest. Strategy ratings can, therefore, tap into stable properties of individual task trials, providing an experimental approach to explore component processes. We adopted such an approach here to functionally dissociate multiple juxtaposed networks that were measured within individual participants and also to provide insight into each network’s functional contributions to task processing.

## METHODS

### Overview

Data for analyses came from previously collected neuroimaging participants (26) paired with newly collected behavioral data that measured the idiosyncratic processing demands of each trial. The task neuroimaging data included 180 trials where participants answered unique questions by selecting one of three possible choices. In the present work, independent online behavioral participants rated the strategies they used to answer each of the 180 questions (Table 1). The strategy ratings were remarkably stable between independent behavioral samples and could be clustered into intercorrelated composites. Functional properties of the networks were examined by asking whether activity levels in distinct brain networks preferentially tracked strategy composite scores.

**Table 1.** The response strategies scale included 16 strategy probes

#	Abbreviation	Strategy Probe	Mean Rating	SD Rating	Interexperiment Reliability (*r*)
1	Significance	To what extent did this question … ask about a matter that is significant to you?	3.18	1.60	0.92
2	Pers_Feelings	… lead you to think about your own preferences, feelings or emotions?	3.62	1.59	0.93
3	Emotions	… evoke any emotion (e.g., happiness, sadness, excitement, etc.)?	3.01	1.62	0.91
4	Pers_Past_Exper	… rely on personal past experiences?	4.15	1.71	0.93
5	Sequence_Events	… lead you to imagine a sequence of events unfolding in your mind?	3.61	1.86	0.91
6	Loc_Obj_Places	… lead you to envision the location of objects or places mentioned in it?	3.70	1.98	0.89
7	Loc_People	… lead you to envision the physical locations of other people?	2.83	1.82	0.86
8	Others_Feelings	… lead you to speculate about the preferences, emotions, or thoughts of other people?	2.90	1.75	0.92
9	Moral_Principles	… make you consider general moral principles?	1.89	1.25	0.91
10	Relationships	… require you to think about the nature of your relationships to other people?	2.16	1.30	0.96
11	Others_Personality	… lead you to consider the personality traits or appearances of other people?	2.27	1.58	0.88
12	Visual_Imagery	… evoke visual imagery in your mind?	4.29	1.89	0.81
13	Recent_Thoughts	… evoke thoughts that have been on your mind recently?	2.49	1.53	0.91
14	Difficulty	When answering this question … how hard did you have to think? (1—thoughts were spontaneous or easy, 7—thoughts were deliberate or difficult)	3.02	1.65	0.93
15	Facts	… to what extent did you rely on facts, as opposed to subjective experiences? (1—relied only on subjective experiences, 7—relied only on facts)	3.85	1.95	0.86
16	Specificity	… how specific were your thoughts? (1—thoughts were broad or general, 7—thoughts were detailed and specific)	4.21	1.91	0.66

Notes: Two phrases (“to what extent did this question…” and “when answering this question…”) were each followed by multiple probes. All probes included a 7-point scale (for probes 1–13: 1—none, 7—a lot; for 14–16: scales shown in the table). Strategy probes 1–8, 14 and 15 were taken or adapted from Andrews–Hanna et al. (23; see their Table S3). For each probe, rating means and standard deviations (SD) are shown, calculated across raters in *exp 1* and *exp 2*. Interexperiment reliability shows the Pearson’s correlation between mean ratings in *exp 1* and *exp 2*, across all trials, for each probe.

### Experiment 1: Initial Exploration of Strategy Ratings

#### Participants.

One hundred and seventy-five paid participants aged 20–28 were recruited from Amazon Mechanical Turk using Cloud Research (45) to answer trial questions and complete surveys about their strategy use while answering the questions. Respondents as young as 18 yr old were included. Participants were English speakers (i.e., native or learned before age 7) within the United States who had high ratings for completion of prior studies as vetted by Cloud Research (90%+ approval rating for at least 100 prior tasks). Each participant provided informed consent online; study protocols were approved by the Institutional Review Board (IRB) of Harvard University. Participants were paid, based on survey piloting, at an estimated rate of at least $10/h. Question and strategy probe forms were administered using Harvard’s Qualtrics platform.

#### Question and strategy probe format.

Online participants answered questions from the episodic projection task of DiNicola et al. (26). Prior to starting, participants were asked to attest that they would “focus exclusively on the survey while participating.” Participants then completed online consent and demographics forms. During instructions, participants practiced by answering one question and then rating how they chose their response, scoring across 16 strategy probes designed to tap into a variety of possible processing components (Table 1). These probes were presented all at once, without instructions about interpretation. During the survey, after each individual question, participants scored these 16 strategies. Given the burden of rating many strategy probes, each participant answered only one subset of the original trial questions (34–38 total trials per participant). The trials were rotated across participants so that 25 participants rated strategies used for each unique trial. As in the original task, each trial asked a question about either a real-world experience or general knowledge and featured three answer choices (see Ref. 26). A few trials required minor wording changes for generalization to online participants (e.g., changing “a trip out of Boston” to “a trip out of your town”). Finally, as a control procedure, a subset of trials was repeated, including six shown to all participants (each from a different condition) to test for potential cohort effects, and with two questions as attention checks (i.e., one targeting task focus and another probing whether participants were reading carefully).

Trial timing was as follows: participants first saw the question and answer options and were asked to respond within 10 s (similar to the neuroimaging protocol). Participants could proceed after responding. After 10 s, they received a reminder to select a response. After answering the question, the participants were then presented with the response strategies scale (RSS; Table 1) and asked to rate their use of 16 strategies on a scale from 1 to 7. The RSS expanded upon the scale used by Andrews-Hanna et al. (see Table S3 in Ref. 23) to incorporate previously assessed strategies (e.g., reliance on memory, personal significance, effort), as well as new strategies, informed by work probing mind-wandering and memory components (e.g., consideration of people’s attributes, moral principles, and relationships; e.g., Refs. 46–49; see also Ref. 50).

#### Exclusion criteria and quality control.

As is often observed in online experimentation, participant compliance varied. A series of quality control (QC) criteria were adopted to conservatively include only participants who fully engaged the task. The criteria were applied before any analysis of factors or assessments of reliability. Participants were excluded if they *1*) spent less than 20 min on the survey, *2*) reported being outside of our age range (18–28 yr old), *3*) had no mouse clicks registered for multiple trial responses (indicating potential automation; 51), *4*) showed clear stereotyped patterns of responding across trials, or *5*) missed more than one check question. Participants were also flagged if they did not comment or write “None” (as requested) in a final feedback box, or if they missed any check questions (i.e., they did not select “fully focused,” did not choose “cats and dogs” as popular pets, or did not respond as “reading this question” on a question included specifically as a compliance check). A single flag (e.g., selecting “somewhat focused” or not writing “None”) did not result in exclusion if no patterned responding or other flags were noted.

Assessment of response patterns was particularly vital to quality control. Thirteen strategy probe ratings for 11 trials (7 that appeared across multiple surveys and 4 unique to a survey) were visualized for each participant by two independent experimenters (LMD and OIA). Strategies were unlabeled during visualization to prevent experimenter bias. A participant was flagged for exclusion if strategy ratings did not differ across or within each trial, or if trials became uniform near the end of a survey. For example, a subset of participants chose a single value for all strategies within each trial (i.e., straight lines in the trial plots), a subset selected values in a clearly stereotyped pattern (e.g., 1-2-3-4-3-2-1-2-3-4 ratings across strategies), and a subset showed evidence of a drop off in performance (with a single rating given to all strategies only in later trials). For participants flagged due to stereotyped responses, all trials were visualized as an additional check.

As a final quality control check, ratings across strategies for the five trials consistently included across all participants were visualized to explore cohort differences. Strategy ratings for each trial showed similar patterns. A linear model testing for rating differences found no significant cohort effects [*F*(6, 665) = 0.36, *P* = 0.90), supporting that a trial’s strategy pattern represented stable properties, rather than idiosyncratic aspects of the rating group. As the replication experiment will reveal, these conservative procedures yielded highly stable estimates of strategy use across independent groups of participants.

One hundred and thirty-six total participants aged 18–25 remained (77.7%) after exclusion (Table 2). Included participants [mean age = 22.7 yr (SD = 1.9 yr), 49% identifying as female] had a mean completion time ranging from 38.2 to 56.7 min across the 7 cohorts (mean = 45.2 min).

**Table 2.** Summary of exclusions in experiment 1

Cohort	# Enrolled	# Exc: Time <20 min	# Exc: Click Count = 0	# Exc: Missed > 1 Check Q	# Exc: Patterned Responses	# Exc: Age	Total Included (%)	Mean Age	# Female
1	25	4*	1	0	2	0	18 (72%)	23.0	7 (39%)
2	25	4	0	1	3	0	17 (68%)	22.8	6 (35%)
3	25	2	0	0	5	0	18 (72%)	22.2	10 (56%)
4	25	0	0	0	3	0	22 (88%)	22.7	14 (64%)
5	25	1	0	1	2	0	21 (84%)	23.0	11 (52%)
6	25	0	1	0	2	1	21 (84%)	22.6	12 (57%)
7	25	2	0	0	4	0	19 (76%)	22.5	7 (37%)
Overall	175	13	2	2	21	1	136 (78%)	22.7	67 (49%)

Notes: 78% of participants were included in analyses. *In *cohort 1*, one participant who took exactly 20 min was excluded. Patterned responding (assessed by 2 independent raters) resulted in the greatest number of exclusions overall. Participants were first excluded if outside of our age range. If participants then fell into more than one category (e.g., <20 min and patterned), they were categorized within the first relevant column, from left to right. Exc, excluded.

#### Strategy clustering.

Strategy clusters were identified with the goal of constructing robust composite scores for subsequent functional network analysis. We first calculated average ratings for each strategy, across respondents and for each unique trial. We *z*-scored ratings within strategies, creating a matrix featuring 16 mean strategy ratings × 180 trials. The raw correlation structure was estimated and visualized, and then hierarchical clustering was used to estimate strategy groupings (*hclust* function and ward.D2 amalgamation procedure in R v3.5.1). We chose hierarchical clustering for the ease of visualizing relations across variables (see also Ref. 48).

### Experiment 2: Prospective Replication of Strategy Rating Structure

*Exp 2* was conducted to examine the stability of online strategy ratings across trials, as well as to replicate the strategy clusters observed in *Exp 1* in an even larger sample, before network exploration in the neuroimaging data.

#### Participants.

Three hundred paid participants aged 20–28 were again recruited from Amazon Mechanical Turk using Cloud Research (45). Respondents as young as 18 were included. Participants were English speakers within the United States who had high ratings for completion of prior studies as vetted by Cloud Research (90%+ approval rating with at least 100 prior tasks approved). Each participant provided informed consent online; study protocols were approved by the Institutional Review Board (IRB) of Harvard University. Question and strategy probe forms were administered using Harvard’s Qualtrics platform.

#### Question and strategy probe format, exclusion criteria, and quality control.

*Exp 2* followed the same procedures for data acquisition, QC, and clustering as in *exp 1*, but with 300 total participants yielding 50 participants rating each unique trial question. Each participant received a subset of 38 total trial questions taken from the episodic projection task’s set of 180 trials. After QC exclusion, 238 participants aged 18–25 remained (79.3%; see Table 3) [mean age = 22.5 yr (SD = 1.9 yr), 61% identifying as female] with a completion time ranging from 38.1 to 46.6 min across the 6 cohorts (mean = 42.5 min). Analysis of strategy ratings across the same subset of six trials as in *exp 1*, repeated for all subjects, again revealed similar patterns and no cohort effect [*F*(5, 570 = 0.26, *P* = 0.94].

**Table 3.** Summary of participant exclusions in experiment 2

Cohort	# Enrolled	# Exc: Time <20 Min	# Exc: Click Count = 0	# Exc: Missed > 1 Check Q	# Exc: Patterned Responses	# Exc: Age	Total Included (%)	Mean Age	# Female
1	50	3	1	1	8	0	37 (74%)	22.9	21 (57%)
2	50	5	0	0	6	0	39 (78%)	22.3	26 (67%)
3	50	5	0	1	3	1	40 (80%)	22.4	18 (45%)
4	50	4	0	1	3	0	42 (84%)	22.5	25 (60%)
5	50	2	1	0	6	0	*40 (80%)	22.6	27 (68%)
6	50	3	0	1	4	2	40 (80%)	22.3	29 (73%)
Overall	300	22	2	4	30	3	238 (79%)	22.5	146 (61%)

Notes: 79% of participants were included in analyses. Patterned responding again resulted in the greatest number of exclusions overall. *In *cohort 5*, one individual was excluded due to reported aphantasia (i.e., inability to represent visual imagery). As in *exp 1*, participants were first excluded if outside of our age range. Exclusions were then categorized within the first relevant column, from left to right. Exc, excluded.

#### Quantifying behavioral rating stability across Experiments.

To examine the stability of measures derived from behavioral strategy probes, we compared the strategy patterns, for each trial, across independent groups of respondents from *exp 1* and *exp 2*. For each trial, we plotted the mean (and standard error) across all 16 strategies from the RSS and calculated trial-level interexperiment reliability (correlations across mean strategy ratings for that unique trial). Varying numbers of participants contributed to the mean ratings across trials, depending on the final participants retained after blind QC for compliance. For nonrepeated trials, the number of respondents estimating strategy use for each unique trial was no fewer than 17 in *exp 1* (mean = 19.4 across cohorts, max = 22; see Table 2) and no fewer than 37 in *exp 2* (mean = 39.7 across cohorts, max = 42, see Table 3). In each experiment, 2–6 trials were repeated for subsets of cohorts (*n*_*exp1* _= 99 or 117, *n_exp2_* = 159 or 198), and at least 6 additional trials across all cohorts (*n_exp1_*_ _= 136, *n_exp2_* = 238).

In addition, to quantify whether a strategy probe was rated similarly, across trials, from one experiment to the next, we calculated probe-level interexperiment reliability. For each probe, we first calculated the mean ratings (and SD) across all 180 trials within each experiment. We then correlated the means across experiments (reported in Table 1).

#### Composite score creation.

With few exceptions, the strategies clustered similarly in *exp 1* and *exp 2* (discussed in detail in the results). Preserving clusters with the strongest between-strategy relations yielded five strategy composites, labeled heuristically as difficulty, scene construction, others-relevant, autobiographical, and self-relevant. Using data from *exp 2*, for each trial, the *z-*normed mean ratings for relevant strategies were summed to produce composite scores (as in Ref. 23).^2^ Strategies that were inconsistent or weakly associated between the two experiments were excluded (“Relationships,” “Specificity,” “Moral_Principles,” and “Loc_People”). In this way, composites represented strategies showing high correlations across multiple independent experiments.

#### Validating composite scores against trial response times.

Strategy composite scores were based on self-reported strategy use. Although reliability could be established for trials, probe questions, and composites, a specific challenge of our approach is validation. As will be seen in the results, one behavioral composite emerged that reflected trial difficulty, creating an opportunity to test the validity of subjective ratings using response time (RT). For each trial, a mean RT was calculated, after exclusion of rare outlier trials with an RT greater than 60 s (0.17% of all trials). These objective RT measures of trial difficulty were compared with the composite scores derived from self-reported strategy use.

### Experiment 3: Examining Strategy-Network Relations

*Exp 3* examined trial-level variation in functional MRI responses to explore relations to trial-level strategy composite scores. The MRI data were included in a prior report by DiNicola et al. (26) analyzed at the level of condition contrasts. Here, the data were reanalyzed at the level of individual trials in the context of the novel behavioral data from *exp 1* and *exp 2*.

#### Participants.

Ten paid adult participants aged 18–25 [mean age = 20.5 yr (SD = 2.1), 9 right-handed, 8 identifying as female] were recruited from the Boston area to complete 4 neuroimaging sessions each. All participants provided informed consent online; study protocols were approved by the Harvard University IRB. Each neuroimaging session featured a battery of tasks, including fixation and the episodic projection task. Full details of task acquisition and preprocessing parameters are provided in DiNicola et al. (26; see also Ref. 52). Only participants who completed all runs of the expanded episodic projection tasks were included in the current study (Exp. 2 and Exp. 3 in Ref. 26). Two individuals who completed only two scanning sessions were excluded (S9 and S13 in Ref. 26).

#### Fixation and episodic projection task paradigms.

Each participant completed 11 runs of a passive fixation task, for intrinsic functional connectivity analysis (7 min 2 s each, 77 m total), as well as 6 runs of an episodic projection task (10 min 12 s each, 61 min 12 s total). Additional tasks were included in the neuroimaging battery, not discussed here (e.g., Refs. 26, 52).

During fixation, participants were instructed to fixate a black plus sign on a light gray background, while remaining alert and still. Fixation runs were intermixed with runs of other tasks, and fixation data were used for functional connectivity analysis to estimate network organization. Critically, networks were identified within each individual independently from (and before) other task analyses and without examination of any of the trial-variation effects explored in this paper.

During each run of the episodic projection task, participants responded to trials varying in self-relevance (Self vs. Non-Self) and temporal orientation (Past, Present, or Future). Crossing of these two dimensions (2 × 3) yielded 6 target conditions of 30 trials each (e.g., Past Self, Past Non-Self, and so on for Present and Future timeframes; 26, see also Ref. 23). Five trials from each condition were presented per task run. Across the 6 runs, each participant performed all 180 unique trials. Participants were instructed to carefully consider the details of each trial’s question before selecting a response (10 s trial, 10 s ISI).

#### MRI data acquisition and processing.

Data were acquired at the Harvard Center for Brain Science using a 3 T Siemens Prisma-fit MRI scanner and a 64-channel phased-array head-neck coil (Siemens Healthcare, Erlangen, Germany). During each scan session, a rapid T1-weighted structural image was acquired, using a multi-echo magnetization prepared rapid acquisition gradient echo (ME-MPRAGE, 53) sequence (1.2 mm isotropic voxels, TR = 2,200 ms, TE = 1.57, 3.39, 5.21, 7.03 ms, TI = 1,100 ms, 176 slices, flip angle = 7°, matrix = 192 × 192 × 176, in-plane GRAPPA acceleration = 4). Blood oxygenation level-dependent (BOLD) data were acquired using a multiband gradient-echo echo-planar pulse sequence (see Refs. 54–57), provided by the Center for Magnetic Resonance Research at the University of Minnesota (2.4 mm isotropic voxels, TR = 1,000 ms, TE = 32.6 ms, flip-angle = 64°, matrix = 88 × 88, 65 slices covering cerebral cortex and cerebellum). All data were processed using a custom analysis pipeline (termed “iProc”; see Refs. 25, 26), designed to preserve within-individual details. Briefly, each participant’s data were registered to a subject-specific, 1-mm isotropic T1 template through a single interpolation, which combined matrices for motion, field map unwarping, alignment to a mean BOLD image, and then to the T1 template.

For functional connectivity analysis, nuisance variables (6 motion parameters and whole brain, ventricular and white matter signals, along with their temporal derivatives) were regressed from the T1-aligned BOLD fixation data. These data were then bandpass filtered at 0.01–0.10 Hz (using AFNI v2016.09.04.1341; 58, 59). For episodic projection task analyses, the whole brain signal was regressed from the T1-aligned task data (see Ref. 26).^3^ All BOLD data from both the fixation task and episodic projection task were then resampled to the fsaverage6 cortical surface mesh (using trilinear interpolation; 60) and smoothed using a 2-mm full-width at half-maximum kernel.

Data were examined for quality. Run-level exclusion criteria included *1*) maximum absolute motion greater than 1.8 mm, *2*) slice-based signal-to-noise ratio less than or equal to 135 (as in Ref. 61), and, for the episodic projection tasks, *3*) eyes closed during skipped task trials. Sixteen fixation runs were excluded across the participants, and one run was included for subject 6, in error, despite maximum motion higher than 1.8 mm. No episodic projection task runs were excluded (see Ref. 26 for behavioral performance).

Functional connectivity (FC) analyses were conducted on fixation data, within each individual, to precisely estimate whole brain network organization. *k-*means estimates of networks were used, as reported in a study by Braga et al. (52). Briefly, for each individual, medial wall vertices were removed, and then time series data from the fixation runs were *z*-normalized, concatenated, and input to the *k*-means algorithm, using default parameters (MATLAB v2015b). Networks were identified within the whole brain *k*-means outputs based on referential features (e.g., Ref. 20). Parcellations were computed while varying *k* from 10 to 20, and the solution featuring the fewest clusters differentiating 6 networks was chosen for each individual (52). These networks included default network A (DN-A) and B (DN-B), a language network (LANG), frontoparietal control network A (FPN-A) and B (FPN-B), and a cingulo-opercular network (CING-OPER).^4^ For two individuals, features of one network were observed in two clusters, which collectively better matched seed-based post hoc checks of the networks. Both were included in the network estimate (FPN-A for S10 and FPN-B for S3), before task analysis (52). The networks were determined fully before the task functional MRI data were examined to avoid any potential bias.

For the episodic projection task, run-specific GLMs were created featuring separate regressors for every trial (e.g., Ref. 64), producing trial-specific beta-maps (see full details in Ref. 26; GLMs created using FSL v5.0.4). Beta values within each network were then extracted and averaged (i.e., across all network vertices), yielding trial-specific responses for each separate network within each participant (180 total trials).

As the current analyses aimed to explore trial-level variation (i.e., differences in stable trial-level properties), after estimating average values for each network within individuals, we averaged trial values across individuals, producing a single network estimate for every unique trial. In this way, within-individual network definition allowed for estimates of network activity that were fully constructed within the idiosyncratic anatomy of each individual. At the same time, stable functional MRI estimates for each of the 180 individual trials were obtained because each trial estimate was the average across the 10 participants’ individualized networks.

#### Examining strategy-network relations.

Each of the 180 trials from our episodic projection task was paired with five mean strategy composite scores (from the online data) and six mean network activity values (from the neuroimaging data). We asked whether variation in our behavioral composite scores, across trials, related to variation in network activity. As a first step, we calculated correlations between trial-level behavioral composite scores and mean functional MRI BOLD response estimates from each of the six networks (using *cor.test* from stats v4.1.1 in R). Data from all trials were included, and Pearson’s correlation values were plotted to visualize patterns for each composite.

As will be seen in the results, particularly strong correlations were found between the Scene Construction composite scores and DN-A response and between the Difficulty composite scores and FPN-B response. Building on these correlational results, we next sought to unpack observed strategy-network relations. Scene Construction, for example, appeared to differentiate DN-A from tightly juxtaposed DN-B, as well as other networks. Relations between Scene Construction and DN-A and Difficulty and FPN-B appeared dissociable. We sought to test these observations using multiple methods.

To quantify whether composite scores significantly predicted network activity, multiple regression was used. Each regression model probed whether composite scores explained variance in a specific network’s response. The relative importance (i.e., *R*^2^ contribution of each regressor) for each composite was also calculated (*relaimpo* v2.2–6 in R; 65). Scatterplots allowed for visualization of different composite-network relations, relative to regression lines and across all 180 trials.

Regression models thus provided insight into how much variance in network activity was captured by our composite scores. For the strongest observed correlations, we also sought to contextualize these values as a percent of the explainable variance, the highest *R*^2^ one could expect, constrained by the internal reliability of our data (i.e., given a true correlation of 1 between a network-composite pair; e.g., Ref. 66; see Ref. 67). Toward this goal, we first calculated split-half reliability for the composites and networks, by halving each dataset, creating vectors of mean values for each half, and correlating those values (adjusted by the Spearman-Brown formula). For network estimates, all split-half combinations were used, and for composite estimates, 1,000 split-half samples (featuring the first 36 participants per strategy probe from *exp 2*, with questions that repeated across surveys only represented in the first set of survey respondents). A maximum explainable variance value was estimated as the product of the reliability, which we compared to our estimated *R*^2^ values.

#### Exploring the impact of difficulty.

As will emerge in the results, initial regression models revealed evidence of intercorrelation between a composite score reflecting Difficulty and other composite scores. In post hoc analyses, we directly tested whether Difficulty impacted other composite-network relations by regressing the impact of Difficulty ratings from all other composite scores and plotting residual composite-network correlations.

#### Probing scene construction and difficulty contrasts to verify network dissociations.

Finally, the analyses supported a double-dissociation between composite scores reflecting Scene Construction and Difficulty in relation to activity in brain networks DN-A and FPN-B. As a final stringent test of this discovery, we used the composite scores to create contrast maps based on trials with high and low values on each of these composites. For Difficulty, we identified the 10 trials with the highest and 10 trials with the lowest composite scores. For each individual, we then created a whole brain contrast map and overlaid the border of the individual’s FPN-B, to compare each individual’s contrast map to their specific FPN-B estimate. For Scene Construction, we used the same process, but only included trials originally considered controls (i.e., from Present Self, Past Non-Self, and Future Non-Self conditions). We selected the 10 control trials with the highest and 10 control trials with the lowest Scene Construction composite scores and then visualized resultant contrast maps in relation to each individual’s DN-A estimate.

## RESULTS

### Behavioral Strategy Probe Ratings Capture Stable Trial-to-Trial Variance

Nearly every strategy probe showed high interexperiment reliability (all *r* > 0.80 except Specificity; see Table 1). Within each trial, mean ratings from the independent cohorts were also strikingly similar (mean *r* = 0.94 across trials, *r* > 0.80 for 98% of trials; see Figs. 1 and 2 for examples). These stable patterns across raters provided evidence that trial “traits” could also inform brain network activity (as indirectly estimated by BOLD functional MRI) from the independent neuroimaging sample.

In addition, trials showed trial-to-trial variation, including within originally designed task conditions. Trials designed to target episodic projection, for example, were previously shown, on average, to preferentially recruit DN-A (26). But strategy patterns varied substantially among individual episodic projection trials (see Fig. 1), raising the question of which component dimensions might explain DN-A recruitment. Original control trials (i.e., designed not to require episodic memory or prospection) also showed marked variability (see Fig. 2). The variation went well beyond differences between conditions. For example, while on average, control trials exhibited lower reliance on the personal past than target trials, as intended, multiple control trials also showed high ratings on strategies relevant to mental scenes or events, also observed in target trials’ patterns. These results highlighted the opportunity to leverage trial-level variation toward novel exploration of network processes, beyond condition-level distinctions.

### Five Strategy Composite Scores Are Supported by Hierarchical Clustering

**Figure 3.**
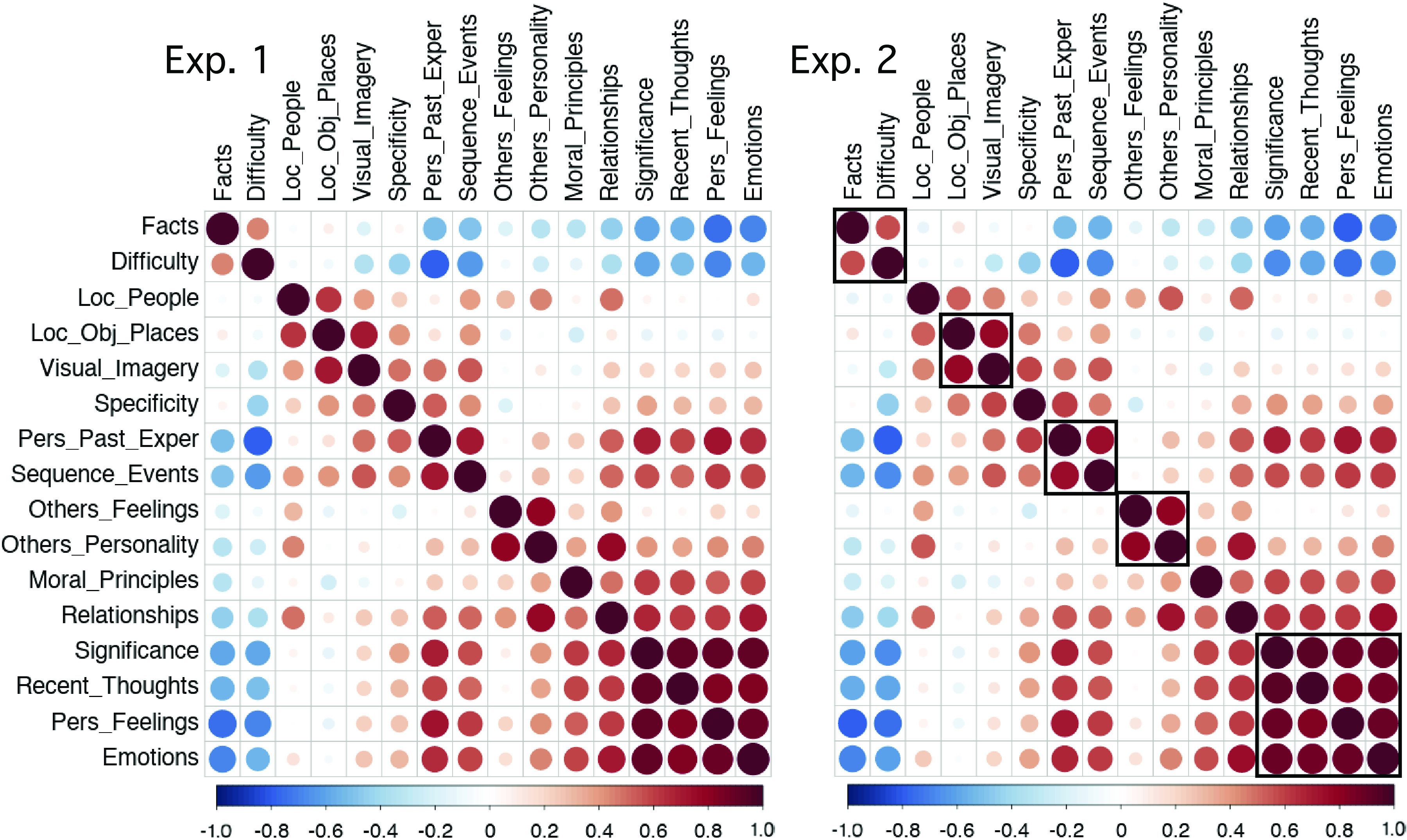
Reliable strategy clusters emerge that capture trial-to-trial variation. *Left*: a correlation matrix illustrates the relations among the 16 scaled strategy probes, using data from all 180 trial questions in behavioral *exp 1*. Strong correlations emerge between subsets of strategy probes indicating that individual trials have distinct rating combinations. For example, trials high in use of visual imagery (Visual_Imagery) also tend to be high in reports of imagining the locations of objects and places (Loc_Obj_Places) and, to a lesser degree, the locations of people (Loc_People). *Right*: an independent correlation matrix from *exp 2* reveals that the strategy relations are reliable (ordered here as in *exp 1* for visualization). The added boxes around correlation clusters in *exp 2* reveal the groupings that were selected based on hierarchical clustering as shown in Fig. 4. Exper, Experiences; Pers, Personal.

To visualize relations among the 16 strategies, we created a correlation matrix, using data from all 180 trials. In *exp 1*, the matrix revealed strong correlations between groups of strategies (Fig. 3, *left*), adding to evidence that trials feature distinct rating combinations. Trials with high ratings for visual imagery (“Visual_Imagery”), for example, were also likely to have high ratings for envisioning the physical locations of objects and places (“Loc_Obj_Places”). Trials with high ratings for considering others’ mental states (“Others_Feelings”) tended also to require imagining others’ personality traits (“Others_Personality”). In *exp 2*, the correlation matrix showed similar structure (Fig. 3, *right*), supporting the reliability of interstrategy correlations.

**Figure 4.**
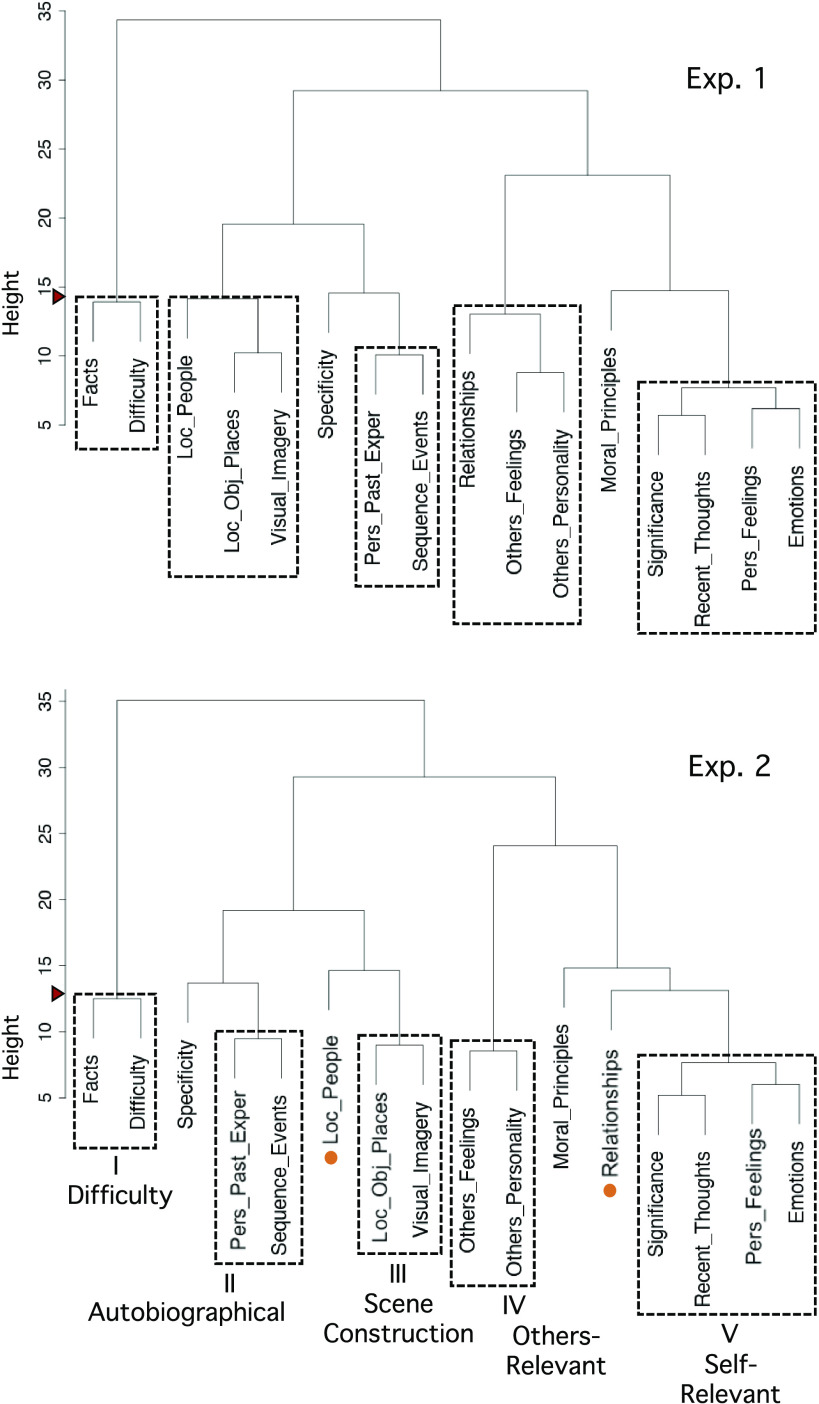
Hierarchical clustering yields five distinct strategy composite scores. Hierarchical clustering identified strategies that could be combined into composite scores for functional network analysis. *Top*: the dendrogram from *exp 1* displays composites using a cut point above “Facts” and “Difficulty,” preserving all clusters at least as strong as this pair. The cut point is noted by a red triangle on the *y*-axis. Dashed boxes show the cluster groupings. *Bottom*: the independently estimated dendrogram from *exp 2* reveals a similar structure. Again using a cut point above “Facts” and “Difficulty” leads to similar clusters that include a core set of strategy probes converged upon across both experiments. These 5 strategy composites were carried forward for analysis of the functional MRI data. They are heuristically labeled as (I) Difficulty, (II) Autobiographical, (III) Scene Construction, (IV) Others-Relevant, and (V) Self-Relevant. Strategy probes that were not consistent between the two experiments (or weakly associated) were not included in the final composite scores, allowing only the most robust and stable strategy probes to be incorporated into the final 5 composite scores.

Using hierarchical clustering, we next identified five strategy groupings that could be combined into composite scores for subsequent network analysis. In *exp 1*, the greatest strategy differentiation appeared for a pair of correlated strategies (“Facts” and “Difficulty”; see Fig. 3, *left*). We cut the clustering dendrogram to preserve clusters at least as strong as this pair, which revealed five groupings (Fig. 4, *top*). In *exp 2*, the independently estimated dendrogram revealed a similar structure. Using the same cut point criterion (above “Facts” and “Difficulty”) produced five strategy groupings that largely matched those from *exp 1*, which we heuristically labeled: (I) Difficulty, (II) Autobiographical, (III) Scene Construction, (IV) Others-Relevant, and (V) Self-Relevant.

The two strategies that were not identically grouped between *exp 1* and *exp 2* (“Loc_People” and “Relationships”), as well as those that showed weaker grouping across both experiments (“Specificity,” “Moral_Principles”), were excluded from composite scores. Out of all strategy probes, “Specificity” also had the lowest interexperiment reliability (*r* = 0.66), and “Moral_Principles” had the lowest mean rating and SD (likely reflecting few morally relevant questions in our task trials; see Table 1).

Given those strategy ratings from *exp 1* and *exp 2* exhibited comparable correlational structure and clustering results, ratings from the larger dataset (*exp 2*) were used to create the strategy composite scores carried forward to analyses of the functional MRI data.

### Difficulty Composite Scores Correlate with Response Times across Trials

**Figure 5.**
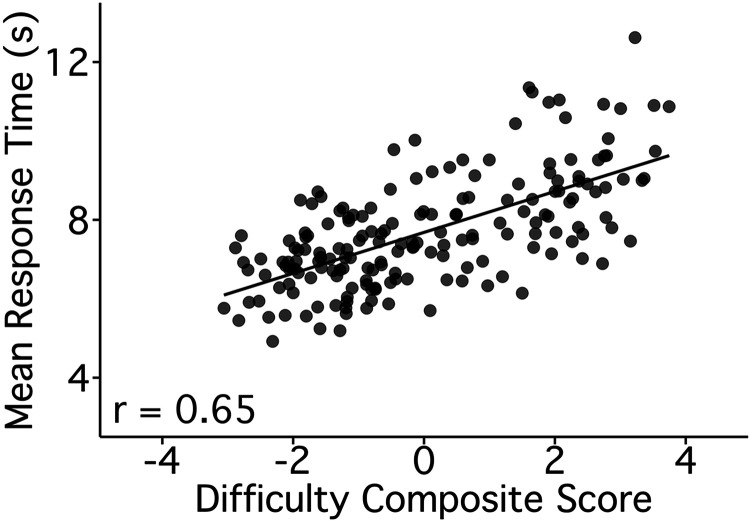
Difficulty composite scores track trial-to-trial variation in response times supporting validity. Response time (RT) estimates provided an opportunity to validate subjective ratings of Difficulty. Mean RTs were calculated for each trial (*y*-axis) and plotted against the Difficulty composite scores (*x*-axis) from *exp 2*. The observed strong positive relation provides evidence for the validity of the Difficulty composite, even though it is based on participant self-report. The Pearson’s correlation value is shown in the bottom left. The line represents a linear model predicting Difficulty scores by RT across trials.

Difficulty composite scores tracked RT values (Fig. 5; *r* = 0.65; CI[0.56, 0.73], *P* < 0.001). Overall, results from *exp 1* and *exp 2* provided evidence that trial-level ratings were stable (Figs. 1 and 2), captured trial-to-trial variation (Figs. 1 and 2), and clustered in reliable ways across experiments (Figs. 3 and 4). For Difficulty (the only possible instance), the composite score was validated against a separate objective measure (Fig. 5). These findings all supported proceeding with analyses of the functional MRI data in relation to strategy composite scores.

### Strategy Composites Scores Correlate Differentially with Network Activity

**Figure 6.**
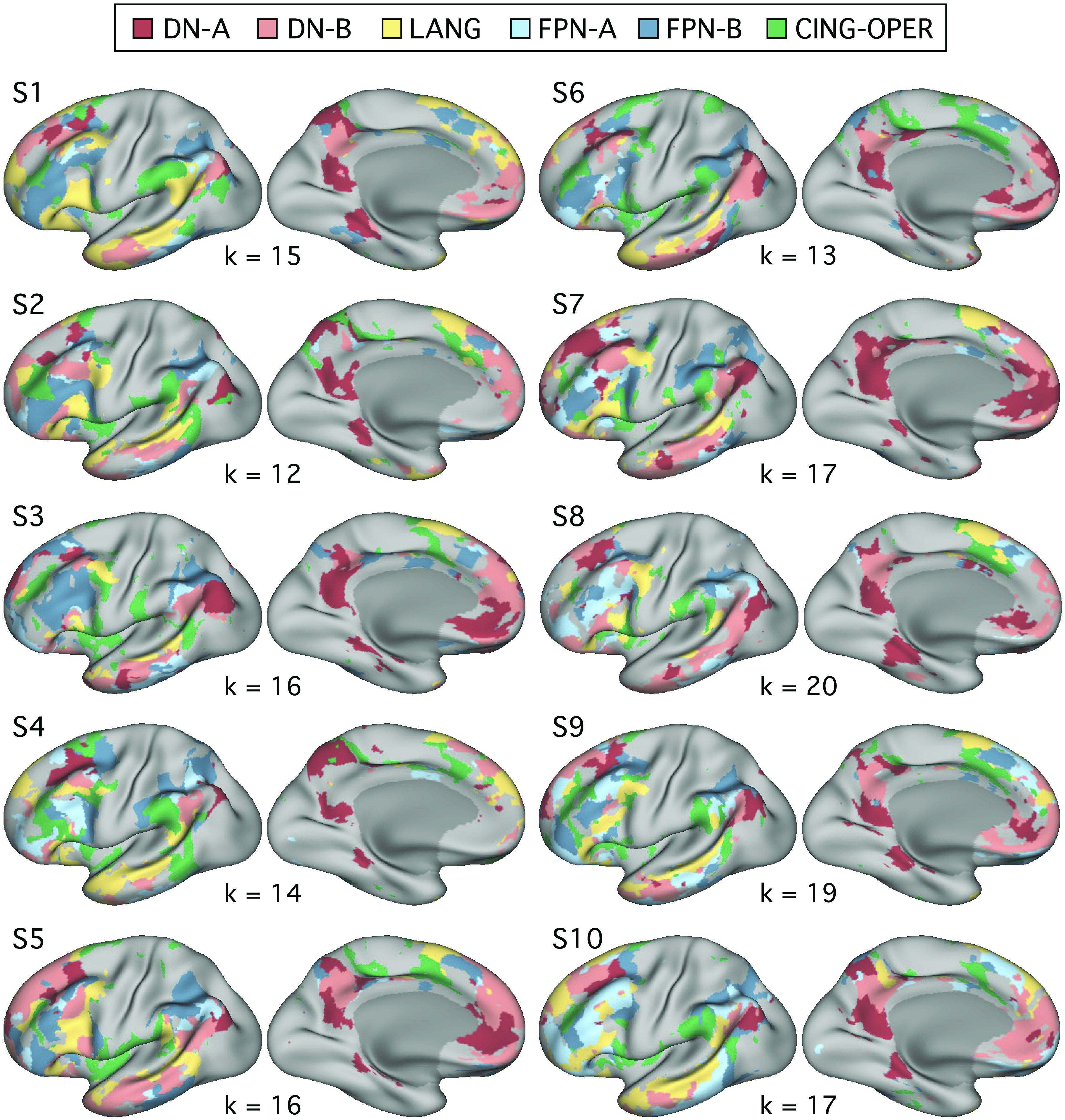
Distributed networks estimated from functional connectivity within individuals: left hemisphere. Whole brain estimates of the 6 networks are displayed for each of the 10 extensively sampled individuals (identical to Ref. 52; see also Ref. 26). For each individual, the *k*-means solution featuring the fewest clusters that differentiated the 6 target networks was chosen. Networks are shown in the left hemisphere and include default network A (DN-A, red), default network B (DN-B, pink), a language network (LANG, yellow), two candidate frontoparietal control networks (FPN-A, light blue; FPN-B, dark blue), and a candidate for a cingulo-opercular network (CING-OPER, green). These networks were defined independently of assessments of functional response properties. Brains in Figs. 6, 7, and 13 are shown at a slightly rotated angle (orientation: *x* = −0.93, *y* = 61.00, *z* = 89.64 in Connectome Workbench v1.3.2; Ref. 68).

**Figure 7.**
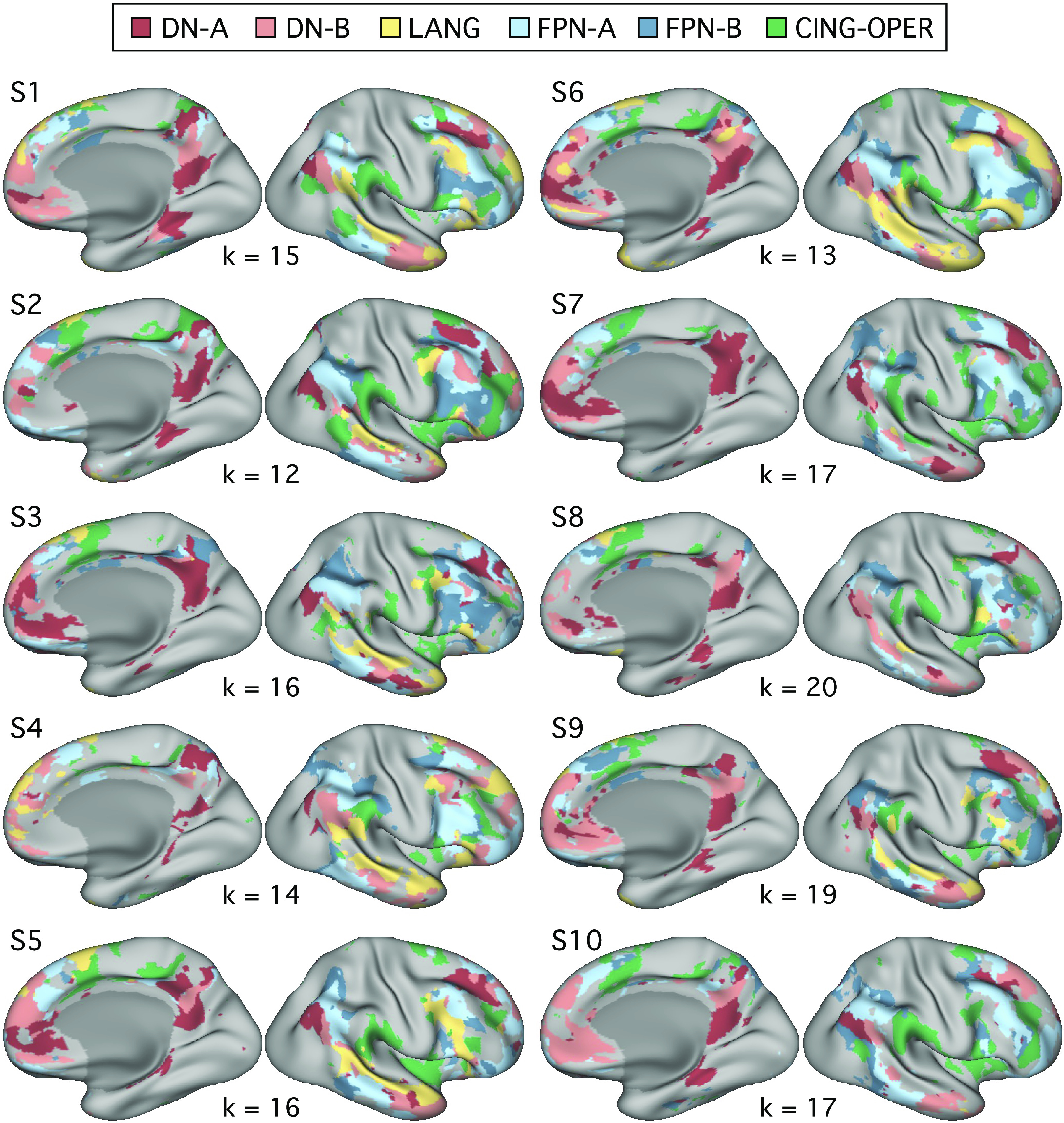
Distributed networks estimated from functional connectivity within individuals: right hemisphere. The networks from Fig. 6 are displayed for the right hemisphere, including default network A (DN-A, red), default network B (DN-B, pink), a language network (LANG, yellow), two candidate frontoparietal control networks (FPN-A, light blue; FPN-B, dark blue), and a candidate for the cingulo-opercular network (CING-OPER, green).

**Figure 8.**
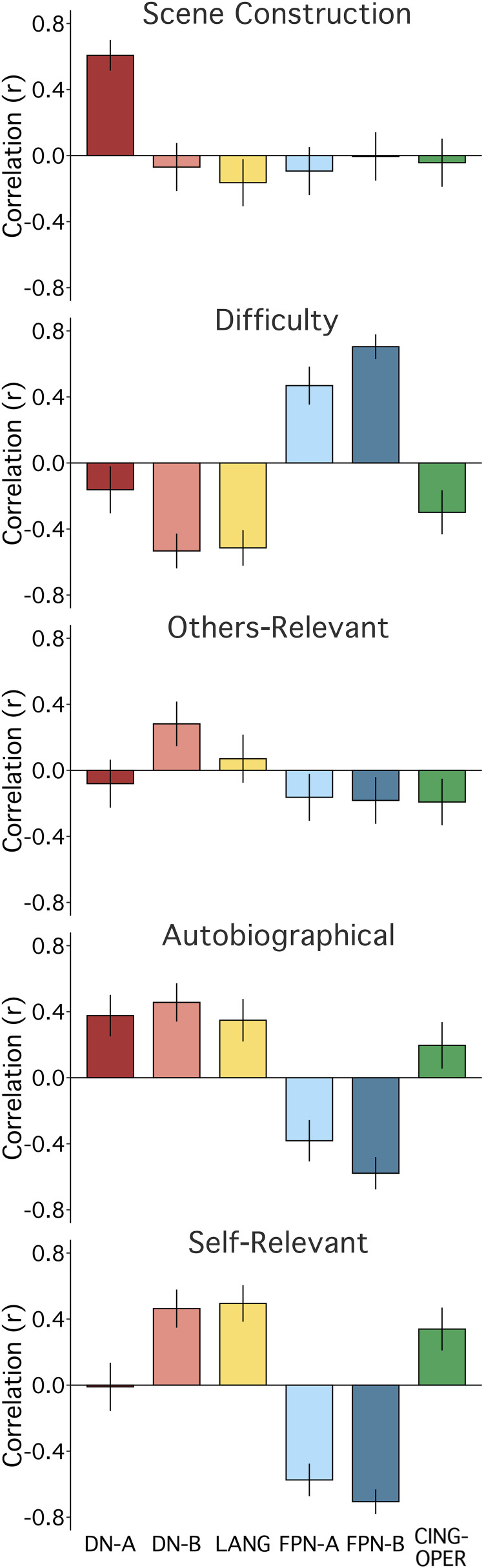
Strategy composites are associated with differential and selective network activity. For each strategy composite, mean scores from all 180 trials were correlated with mean response estimates for each of the 6 networks. The strategy is labeled at the top of each plot; the six colored bars reflect the Pearson’s correlations with 95% confidence intervals. *Scene Construction*: a particularly striking and selective relation is observed between Scene Construction composite scores and DN-A activity. *Difficulty*: the Difficulty composite scores show a strong positive correlation to FPN-B and a strong but weaker relation to FPN-A. *Others-Relevant*: the Others-Relevant composite scores reveal a modest association with DN-B. *Autobiographical and Self-Relevant*: results between the Autobiographical and Self-Relevant composite scores are more ambiguous (partially related to confounding effects of Difficulty; see Fig. 12). Networks, from left to right: default network A (DN-A, red), default network B (DN-B, pink), a language network (LANG, yellow), two candidate frontoparietal control networks (FPN-A, light blue; FPN-B, dark blue), and a candidate for the cingulo-opercular network (CING-OPER, green).

We first calculated correlations between each of the five strategy composites and functional MRI response in each of the six independently estimated networks (Figs. 6 and 7), using data from all 180 trials. Plotting Pearson’s correlation values revealed a particularly striking relation between Scene Construction scores and DN-A activity (Fig. 8). Scene Construction differentiated DN-A from interwoven DN-B and from all four additional networks. DN-B, in turn, showed a selective (albeit weaker) correlation to Others-Relevant scores. In addition, strong correlations were noted between the Difficulty composite score and both FPN-A and FPN-B. More ambiguous network-composite results were revealed for Autobiographical and Self-Relevant scores (due, in part, to confounding effects of Difficulty; see Fig. 12).

To unpack these findings, we first probed Scene Construction’s relation to DN-A and DN-B. The episodic projection task was originally designed to differentiate these parallel networks, using condition-level contrasts (26). The observed correlation suggested that DN-A might preferentially support Scene Construction processes, which would inform differentiation between these tightly juxtaposed networks (e.g., Refs. 9, 26, see also Ref. 21).

### Scene Construction is Selectively Related to DN-A but Not DN-B Activity

**Figure 9.**
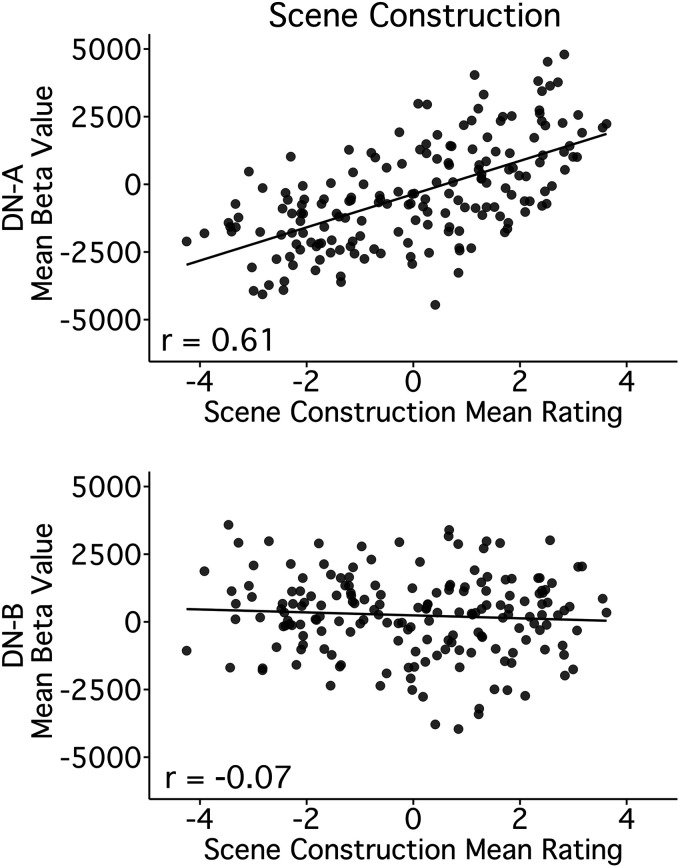
Scene construction is selectively related to DN-A but not DN-B activity. Scatter plots of individual trial activity levels within DN-A illustrate a strong relation to the Scene Construction composite. *Top*: the mean activity level for each of the 180 trials is plotted for DN-A (*y*-axis) against the mean Scene Construction composite scores (*x*-axis). Note that each separate point represents the mean behavioral score for that unique trial from 37–42 participants and the mean functional MRI response for that unique trial averaged across 10 participants. There is a striking linear relationship between the Scene Construction composite and DN-A activity. *Bottom*: the mean activity level for each trial is similarly plotted for the adjacent network DN-B against the same Scene Construction composite scores (*x*-axis). There is minimal relation. Pearson’s correlation values are shown in the bottom left corners. DN-A, default network A; DN-B, default network B.

Comparing Scene Construction scores, across trials, to DN-A activity revealed a strong positive correlation (Fig. 9, *top*; *r* = 0.61, CI [0.51, 0.69], *P* < 0.001). Conversely, DN-B showed a nonsignificant negative correlation to Scene Construction (Fig. 9, *bottom*; *r* = −0.07, CI [−0.21, 0.08], *P* = 0.35).

Multiple regression, with network-specific models, supported the observed dissociation. Of note, initial models included all five strategy composites, but variance inflation factors (VIFs) revealed moderate intercorrelation between Difficulty, Autobiographical, and Self-Relevant scores (VIF > 3; e.g., see Ref. 69). To avoid multicollinearity from a Difficulty confound (which can reduce model accuracy and introduce redundancy), we removed Autobiographical and Self-Relevant composites from subsequent models (see also Fig. 12). Remaining VIF factors suggested no persisting multicollinearity (i.e., equaled 1), so we interpreted regression models featuring Difficulty, Scene Construction, and Others-Relevant composite scores.

For DN-A, this model significantly predicted activity [*F*(3,176) = 36.91, *P* < 0.001], and Scene Construction was the only significant predictor, accounting for most of the variance in DN-A response (*R*^2^_Scene Construction_ = 0.36, *P* < 0.001; *R*^2^_Full Model_ = 0.39). For DN-B, the overall model was significant [(*F*(3,176) = 28.55, *P* < 0.001], but Scene Construction was not a significant predictor and accounted for almost no variance in DN-B response (*R*^2^_Scene Construction_ = 0.01, *P* = 0.06; *R*^2^_Full Model_ = 0.33). These results supported a selective relation between Scene Construction scores and DN-A activity. Although DN-B is tightly juxtaposed to DN-A across the cortical mantle (e.g., Ref. 20), DN-A appears to play a distinct role in Scene Construction processes.

As an additional consideration, the Scene Construction composite included strategies for using visual imagery and for considering the locations of objects or places. A third strategy, for considering the locations of people, clustered with this composite in *exp 1* and was related, but more weakly, in *exp 2*. To test whether excluding this strategy impacted the observed dissociation between DN-A and DN-B, in post hoc analyses, we created a separate composite that included “Visual_Imagery,” “Loc_Obj_Places,” and “Loc_People” ratings. Findings were comparable. For DN-A, this composite (replacing Scene Construction in the original model) was still a significant predictor, again accounting for most of the variance in DN-A response (*R*^2^ = 0.38, *P* < 0.001; *R*^2^_Full Model_ = 0.43; all three predictors now *P* < 0.05). For DN-B, this composite was not a significant predictor (*R*^2^ = 0.00, *P* = 0.26; *R*^2^_Full Model_ = 0.32; both other predictors *P* < 0.01).

### Trial-Level Variation in Scene Construction Tracks DN-A Activity, Including for Control Trials

Given that the episodic projection task was designed to target DN-A activity (26), we next aimed to test whether a link between Scene Construction and DN-A simply recapitulated previous, condition-level results. Namely, in prior work, trials from the Past and Future Self conditions were shown to preferentially recruit DN-A (26). Correlations between DN-A response and Scene Construction scores might predominantly reflect the same condition-level distinctions.

**Figure 10.**
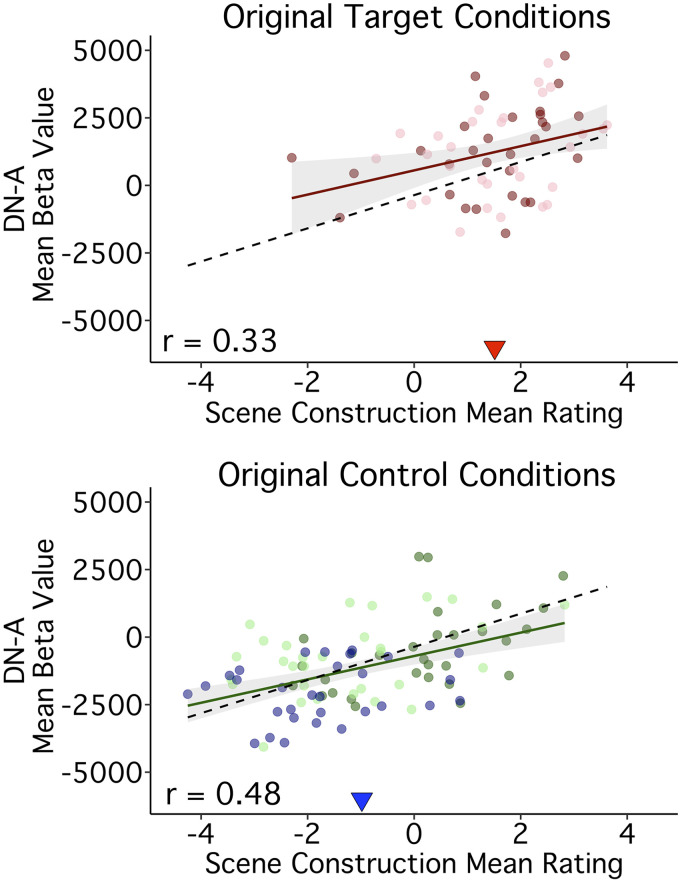
Trial-to-trial variation in Scene Construction tracks DN-A activity levels even for trials that do not involve episodic remembering or prospection. Scatter plots are displayed for DN-A split by whether the trials originated from the target conditions constructed to demand episodic projection (*top*) or were originally included within control conditions constructed to minimize such demands (*bottom*). The correlation with Scene Construction is present in both sets of trials with a strong and clear linear association within the original control trial conditions. Points are colored based on their condition origin: Past Self (pink), Future Self (red), Present Self (blue), Past Non-Self (dark green), and Future Non-Self (light green). Each point represents a single trial; Pearson’s correlation values are shown in the bottom left corners. Regression lines in color include only the trials from target (*top*) or control (*bottom*) conditions; black dashed regression lines include all 180 trials. Triangles indicate the mean composite score across trials in each plot, highlighting condition-level differences. Scene Construction tracks DN-A response both for trials originally constructed to target DN-A and for trials constructed to minimize demands on episodic projection. DN-A, default network A.

We, therefore, ran additional analyses restricted either to those original target trials or to control trials constructed not to include episodic projection demands (from Present Self, Past Non-Self, and Future Non-Self conditions). Correlation results revealed that Scene Construction scores for both the original target (*r* = 0.33, CI[0.08, 0.54], *P* < 0.05) and original control trials (*r* = 0.48, CI[0.30, 0.62], *P* < 0.001) tracked DN-A response (Fig. 10). Although Scene Construction scores were higher, on average, for the target trials as compared with the control trials [*t*(146) = −10.82, *P* < 0.001; see triangles in Fig. 10), Scene Construction ratings were significant predictors of DN-A activity in a model restricted to the control trials and still accounted for the most variance (*R*^2^_Scene Construction_ = 0.19, *P* < 0.001; *R*^2^_Full Model_ = 0.32, Difficulty also *P* < 0.01).

To reiterate, even for trials designed not to require episodic projection, the extent to which trials required scene construction predicted DN-A activity. This provides evidence that a core process subserved by DN-A, regardless of episodic projection, is mental construction of scenes (see also Refs. 5, 33).

### Scene Construction and Difficulty Support a Robust Functional Double Dissociation between DN-A and Another Juxtaposed Network FPN-B

To further examine network heterogeneity, we leveraged the composites with the strongest network correlations, Scene Construction and Difficulty, toward probing potential functional dissociation between DN-A and FPN-B. These networks also feature juxtaposed regions in multiple cortical zones (Figs. 6 and 7; see also Refs. 20, 52).

**Figure 11.**
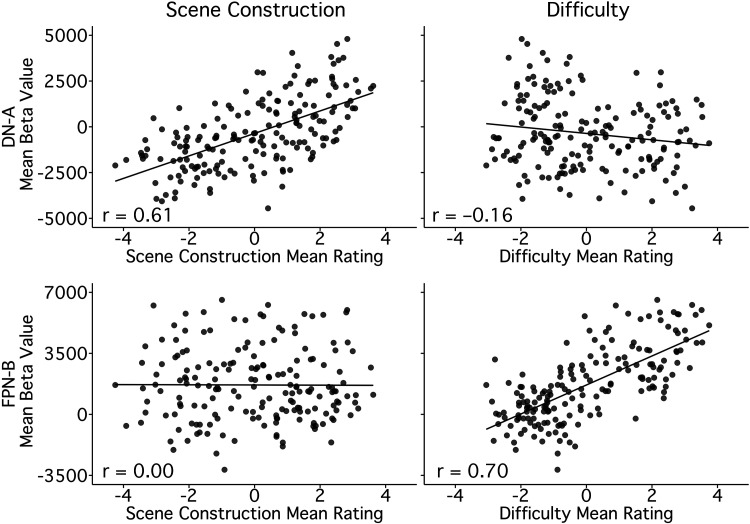
Contrasting Scene Construction and Difficulty reveals a double-dissociation between DN-A and FPN-B. Scatter plots contrast the differential relations of DN-A and FPN-B with the Scene Construction and Difficulty composite scores. *Top, left*: the mean activity level for each of the 180 trials is plotted for DN-A (*y*-axis) against the mean Scene Construction composite scores (*x*-axis). *Bottom, left*: the mean activity level for each trial is plotted for FPN-B (*y*-axis) against the mean Scene Construction composite scores (*x*-axis). Note the absence of a relation. *Top, right*: the mean activity level for each trial is plotted for DN-A (*y*-axis) against the mean Difficulty composite scores (*x*-axis). *Bottom, right*: the mean activity level for each trial is plotted for FPN-B (*y*-axis) against the mean Difficulty composite scores (*x*-axis) revealing a strong, positive relation. Pearson’s correlation values are shown in the bottom left corners. Scene Construction scores track DN-A activity and Difficulty scores track FPN-B activity, but not vice versa, illustrating a functional double-dissociation between these two closely juxtaposed networks. DN-A, default network A; FPN-B, frontoparietal control network B.

As described, Scene Construction scores correlated to DN-A activity (*r* = 0.61, CI [0.51, 0.69], *P* < 0.001). Conversely, DN-A showed a weakly negative correlation to Difficulty scores (*r* = −0.16, CI [−0.30, −0.02], *P* < 0.05), which were strongly associated with activity in the adjacent FPN-B (*r* = 0.70, CI [0.62, 0.77], *P* < 0.001). FPN-B, in turn showed no relation to Scene Construction (*r* = 0.00, CI [−0.15, 0.14], *P* = 0.95), illustrating a functional double dissociation (Fig. 11).

Multiple regression confirmed the double dissociation. In a model featuring Scene Construction, Difficulty, and Others-Relevant scores, Scene Construction accounted for the most variance in DN-A activity across trials and was the only significant predictor, as described above (*R*^2^ = 0.36, *P* < 0.001). For FPN-B, Difficulty was the only significant predictor and accounted for most of the model’s variance in network response (*R*^2^_Difficulty_ = 0.48, *P* < 0.001; *R*^2^_Full Model_ = 0.50).

To better interpret the robustness of these relations, we also calculated the internal reliability of our measures and estimated the proportion of explainable variance for each composite-network pair. The split-half reliability of the composite scores was high (*r*_Scene Construction_ = 0.92, *r*_Difficulty_ = 0.95), as was reliability of the network activity values (*r*_DN-A_ = 0.87, *r*_FPN-B_ = 0.86).^5^ Comparing our models’ *R*^2^ values to the product of these reliability scores, for each composite-network pair (as an estimate of explainable variance) suggested that Scene Construction scores accounted for ∼47% of the explainable reliable variance in DN-A activity, and Difficulty scores for ∼60% in FPN-B activity. Overall, the dissociation between DN-A and FPN-B adds to evidence that parallel association networks, with side-by-side regions across association cortex, can be robustly functionally dissociated (as originally suggested in Ref. 70).

### Strategy Composite Score Correlations after Regression of Difficulty

**Figure 12.**
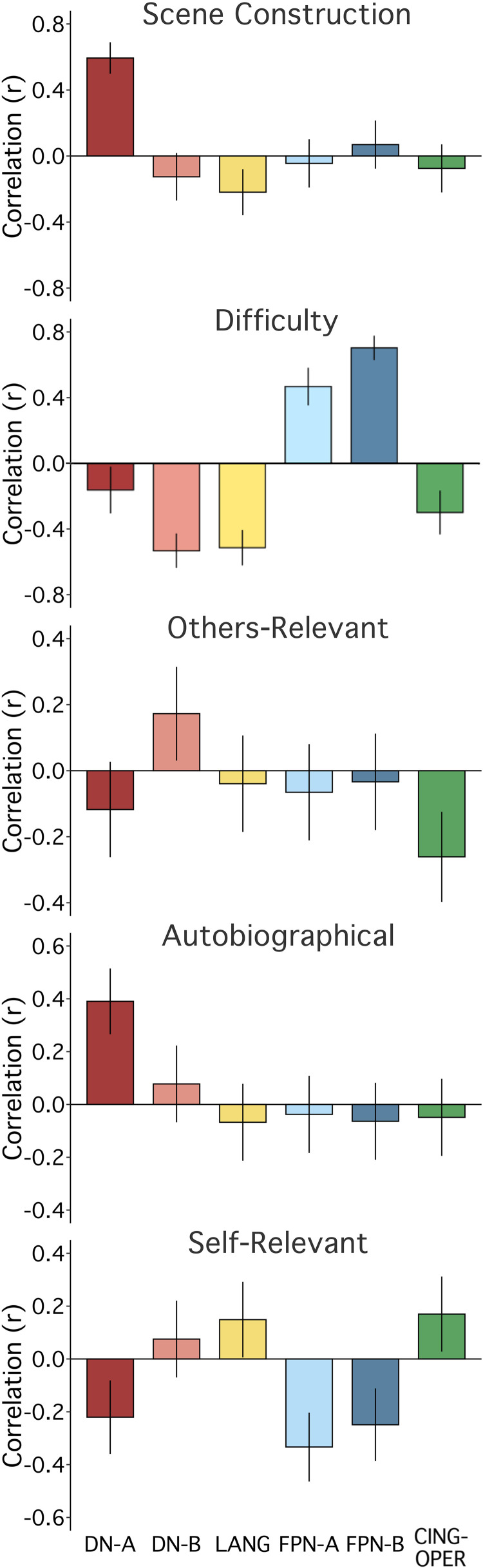
Strategy composites are associated with differential and selective network activity after regression of Difficulty. Given the possibility of a confounding effect of Difficulty on estimates of network selectivity, the network correlation bar plots were recomputed after regressing the Difficulty composite scores (see text). *Scene Construction*: Scene Construction composite scores maintained a strong and selective correlation to DN-A activity. *Difficulty*: as mandated by the analysis, variation related to the Difficulty Composite scores was removed. Since correlations could not be estimated to a vector of zeros, the original Difficulty plot (from Fig. 8, faded here) is shown for reference. *Others-Relevant*: the Others-Relevant composite scores maintained a weaker but selective relation to DN-B activity. *Autobiographical*: of interest, the Autobiographical composite scores, once Difficulty was regressed, revealed a pattern similar but weaker to that of Scene Construction. *Self-Relevant*: the Self-Relevant composite scores were nonspecific. Networks, from left to right: default network A (DN-A, red), default network B (DN-B, pink), a language network (LANG, yellow), two candidate frontoparietal control networks (FPN-A, light blue; FPN-B, dark blue), and the cingulo-opercular network (CING-OPER, green).

Given evidence of a possible confounding effect of Difficulty on multiple composite-network relations, in post hoc analyses, we regressed the Difficulty composite scores from all other composites. Residual data continued to reveal a strong, selective relation between Scene Construction scores and DN-A activity (*r* = 0.59, CI [0.50, 0.68], *P* < 0.001). Effort does not account for Scene Construction’s relation to DN-A response (see Fig. 12, *top*).

Autobiographical scores also showed a selective, positive correlation to DN-A activity after Difficulty regression (*r* = 0.39, CI [0.26, 0.51], *P* < 0.001), and the positive correlation between Scene Construction and Autobiographical composites strengthened. Scene Construction was still the strongest predictor of DN-A response within a model also featuring Others-Relevant, Autobiographical, and Self-Relevant composites (both of which were also significant: *R*^2^_Scene Construction_ = 0.25, *P* < 0.001; *R*^2^_Autobiographical_ = 0.09, *P* < 0.05*;* *R*^2^_Self Relevant_ = 0.05, *P* < 0.001; *R*^2^_Full Model_ = 0.40). We unpacked these results in further post hoc testing (see *Further Tests Do Not Support Contribution of DN-A to Recollection of the Personal Past*).

Others-Relevant scores maintained a weak but selective positive relation to DN-B response after regression of Difficulty (*r* = 0.17, CI [0.03, 0.31], *P* < 0.05). We initially expected a relation between Others-Relevant scores and DN-B response, based on evidence of DN-B recruitment for social functions (e.g., Ref. 26) and ample evidence that relevant regions participate in representing others’ thoughts (e.g., see Refs. 71–73). A model featuring the four remaining composites was significant (*P* < 0.01, with only Self-Relevant as a nonsignificant predictor) but only modestly predicted variance in DN-B activity overall (*R*^2^_Full Model_ = 0.08). The positive relation to Others-Relevant scores accounted for similar variance to a negative relation to Scene Construction (*R*^2^_Others-Relevant,_ = 0.03, *P* < 0.05, *R*^2^_Scene Construction_ = 0.03, *P* < 0.01).

For both Autobiographical and Self-Relevant composites, other observed network correlations did not survive regression of the Difficulty composite scores (compare Fig. 8 to Fig. 12). These results aligned with the evidence of multicollinearity between Difficulty, Autobiographical, and Self-Relevant scores in our initial regression model. Comparing Autobiographical and Self-Relevant scores to mean behavioral RT also supported a Difficulty confound; both composite scores were strongly, negatively correlated with RT (*r* = −0.44 for both, *P* < 0.001). No relation to RT was found for Scene Construction composite scores (*r* = 0.00, *P* = 1.00) and a weaker relation for Others-Relevant scores (*r* = −0.16, *P* < 0.05).

These findings indicate that our data could reduce to three clusters, related to Scene Construction, Others-Relevant, and trial Difficulty dimensions (e.g., with Self-Relevant scores along a continuum of effort). Revisiting our initial composite characterization, both regressing and reverse-coding Difficulty composite strategies (i.e., with higher values for easier and subjective trials) preserved Scene Construction and Others-Relevant correlations, separable from an intercorrelated Self-Relevant cluster.

The relation between Scene Construction scores and DN-A response and the separate relation between Difficulty scores and FPN-B response becomes even more compelling given these additional analyses. As an additional post hoc test, we next probed these two relations to assess whether maps based solely on trial groupings from the composite scores could yield selective network recruitment within individual participants.

### Distinct Networks Can Be Recapitulated within Individuals from Small Numbers of Trials That Differ in Strategy Composite Scores

As a test of the discovery that Scene Construction and Difficulty scores differentiate activity between juxtaposed networks, we created within-individual, whole brain contrast maps using trials with high and low scores on the Scene Construction and Difficulty composites. Importantly, for Scene Construction, we restricted the analysis only to trials from the original control conditions. Thus, this contrast is completely orthogonal to the originally envisioned condition contrasts in DiNicola et al. (26).

**Figure 13.**
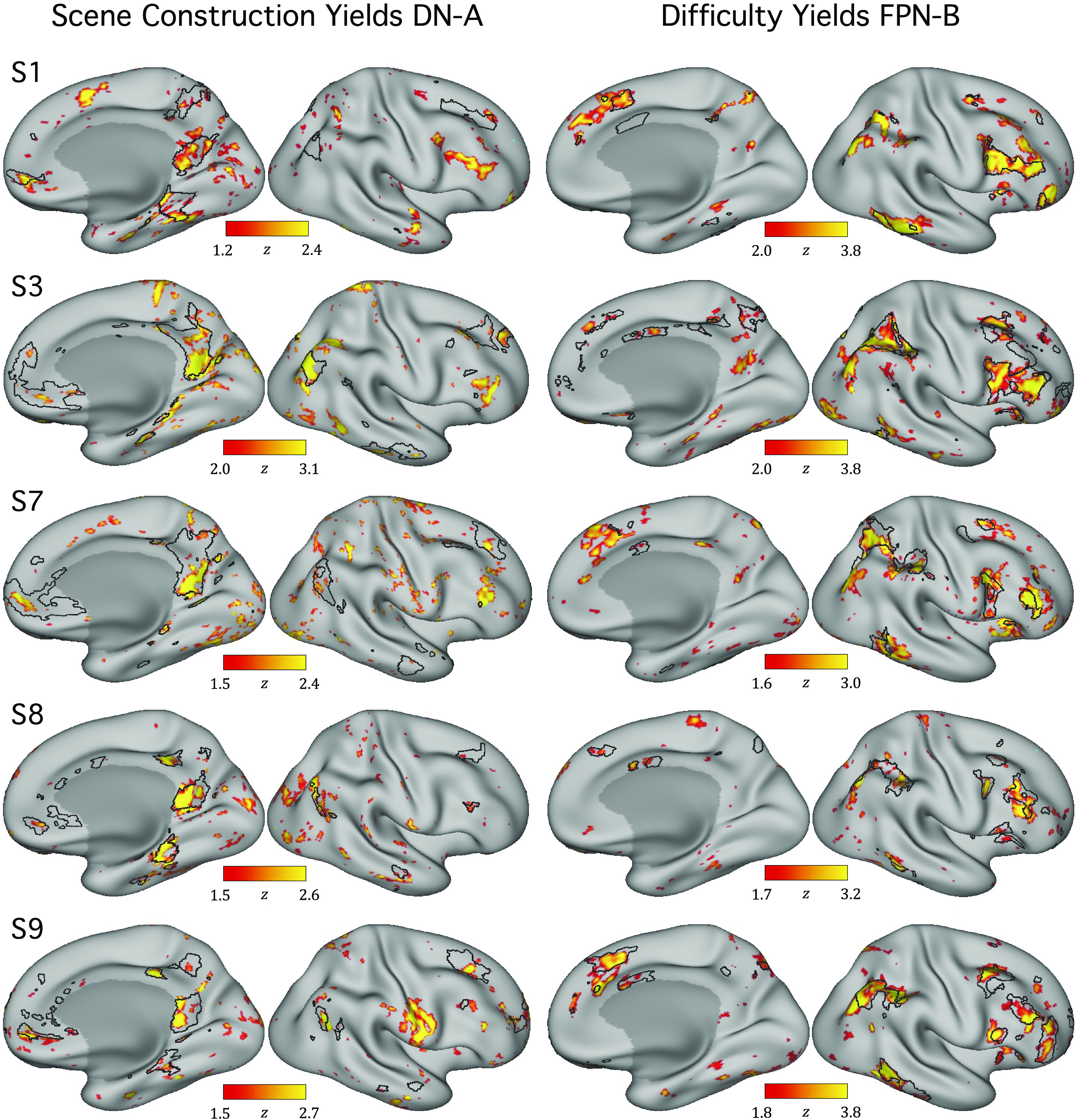
Post hoc contrasts of extreme trials can recapitulate networks within the individual. As a confirmation of the discovery that Scene Construction and Difficulty are associated with functional MRI response levels in distinct networks, within-individual contrast maps are displayed. *Left*: for 5 selected individuals, maps illustrate the contrast of the 10 trials with the lowest Scene Construction composite scores versus the 10 trials with the highest scores, selected only from the original control conditions. Note that even with this relatively small amount of data per individual (from trial conditions originally selected to minimize demands on episodic projection), functional MRI differences emerge across the distributed regions that comprise DN-A. The black outlines show the independently estimated boundaries of DN-A for each participant. *Right*: maps illustrate the contrast of the 10 trials with the highest Difficulty composite scores versus the 10 trials with the lowest scores. Differences emerge that fall within FPN-B. These contrasts were not possible in initial condition-level analyses (26) and provide evidence of novel processing insights. Maps are shown for the right hemisphere. DN-A, default network A; FPN-B, frontoparietal control network B.

Each individual’s estimated DN-A border was overlaid upon the Scene Construction contrast maps. Results revealed alignment between the contrast maps and DN-A (Fig. 13, *left* column). Overlap was observed within midline regions, including in retrosplenial cortex and posterior parahippocampal cortex (PHC), previously linked to Scene Construction ratings (e.g., Ref. 23, see also Ref. 5), as well as in distributed DN-A regions, including in dorsolateral PFC, lateral posterior parietal cortex, and lateral temporal cortex. Although correspondence was not perfect and varied by individual, the noise in these plots was not surprising. Rather, the overlap with DN-A estimates in a subset of participants was striking, given that this contrast included a relatively small number of trials and only those previously treated as controls. The control trials highlighted in this contrast had an average Scene Construction score of 1.91, as compared with 3.01 if we had used the top target trials (see Fig. 10 for the full distributions of Target and Control trials). Thus, even when only considering a weaker subset of trials, and those explicitly designed to minimize demands on episodic projection, contrasting trials with higher versus lower Scene Construction scores reveal activity across the distributed DN-A network.^6^

For Difficulty contrasts, each individual’s estimated FPN-B border was overlaid. Comparing the contrast, within individuals, to network borders again revealed evidence of overlap (Fig. 13, *right* column). In most tested individuals,^7^ Difficulty maps corresponded to FPN-B regions across distributed cortical zones, including in not only prefrontal cortex (as might be expected for cognitive control; e.g., Ref. 74; see also Ref. 75) but also parietal, temporal, and midline zones. The maps provide evidence that FPN-B supports processes related to effortful control and illustrate the power of the described data-driven approach: individually defined networks can be reproduced from process-level dissociations, with contrasts created from independent ratings, not possible in prior condition-level analyses. This strategy is powerful even for dimensions (Difficulty) beyond the task’s original design and in networks (FPN-B) not included in the initial analyses.

### Further Tests Do Not Support Contribution of DN-A to Recollection of the Personal past

In addition to the strong relation between Scene Construction and DN-A, following Difficulty regression, Autobiographical scores showed a selective relation to DN-A response. In additional post hoc tests, we further examined DN-A’s contributions to scene construction and self-reported reliance on mnemonic processes using the Difficulty-regressed data.

**Figure 14.**
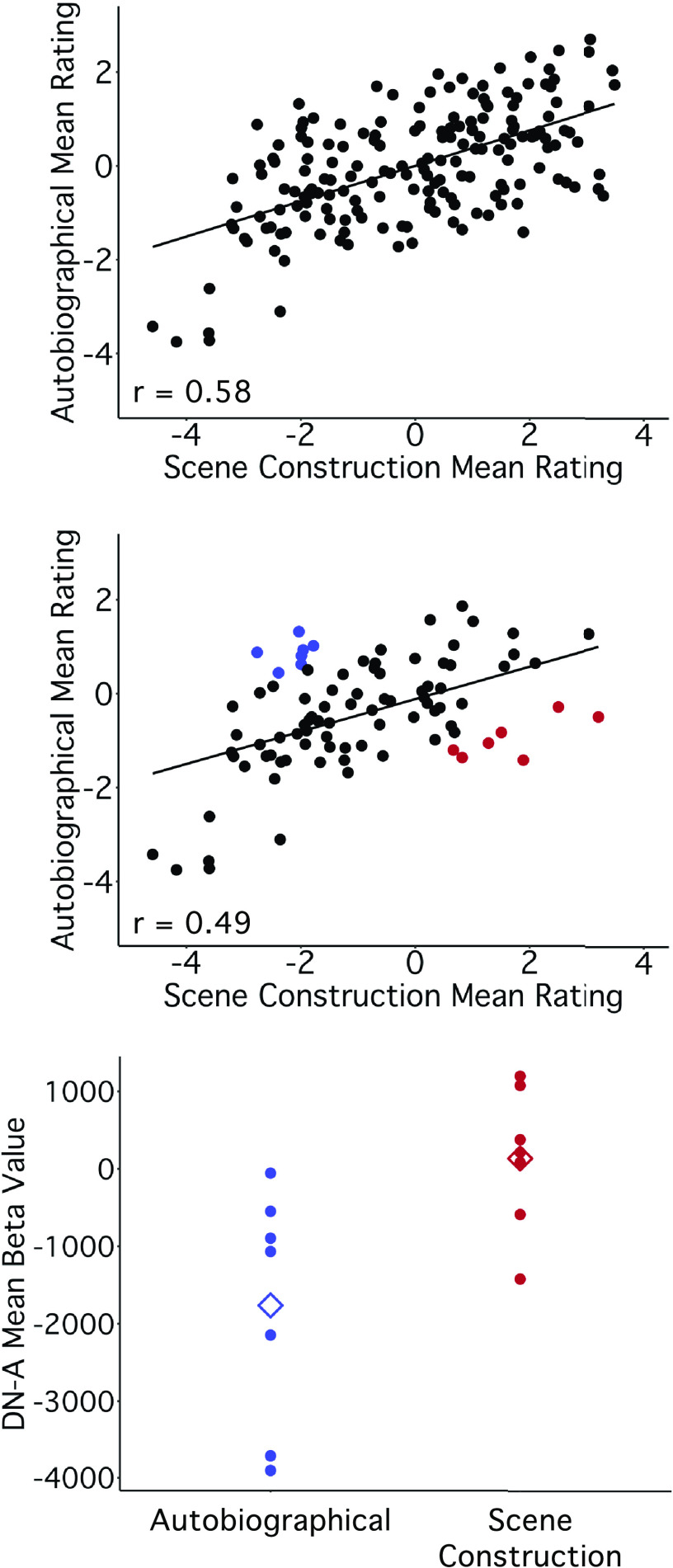
Further exploration of Autobiographical and Scene Construction scores in relation to DN-A response. Following regression of Difficulty, the trial-to-trial correlation between Autobiographical and Scene Construction scores strengthened (*r* = 0.58, *top*). Even for trials originally treated as controls (from Present Self and Past and Future Non-Self conditions), this correlation remained strong (*r* = 0.49, *middle*). But among control trials, subsets of questions were identified that showed higher scores either on the Autobiographical (blue) or the Scene Construction composite (red). Plotting the DN-A response values for each trial in these subsets (*bottom*) revealed higher DN-A activity during trials in the Scene Construction subset. Mean DN-A response in each trial subset is shown by a diamond. DN-A, default network A.

Scene Construction and Autobiographical scores were strongly correlated across all trials (*r* = 0.58, CI[0.48, 0.67]; Fig. 14, *top*) and even across control trials (*r =* 0.49, CI[0.32, 0.64], both *P* < 0.001; Fig. 14, *middle*), presenting a challenge to parsing differences. But among the control trials, subsets showed higher scores on either the Autobiographical or Scene Construction composite (Fig. 14, *middle*). After visualizing the distribution of control trials, we selected a subset representing the 14 most extreme trials (7 higher on Autobiographical scores, clustered in the upper left plot quadrant, and 7 higher on Scene Construction, from the lower right). Examining DN-A response across these trials revealed higher values for the Scene Construction subset [*t*(9.77) = 2.80, *P* < 0.01; Fig. 14, *bottom*].

What is more, unpacking the Autobiographical composite, one strategy probe measured reliance on memory (i.e., Pers_Past_Exper in Table 1), whereas the other measured envisioning a sequence of events (Sequence_Events), also relevant to constructing mental scenes. To assess whether component strategies differentially accounted for variance in DN-A activity, we tested a model featuring each of the Scene Construction and Autobiographical probes in relation to DN-A response (following Difficulty regression).^8^ Within the model (*R*^2^_Full Model_ = 0.50), three predictors were significant, including both Scene Construction probes (*R*^2^_Loc_Obj_Places_ = 0.31, *P* < 0.001, *R*^2^_Visual_Imagery_ = 0.09, *P* < 0.01) and Sequence_Events (*R*^2^ = 0.10, *P* < 0.01). Little variance in DN-A activity was accounted for by the Pers_Past_Exper probe (*R*^2^ = 0.01, *P* = 0.48), the only one specific to recollection of the past.

**Figure 15.**
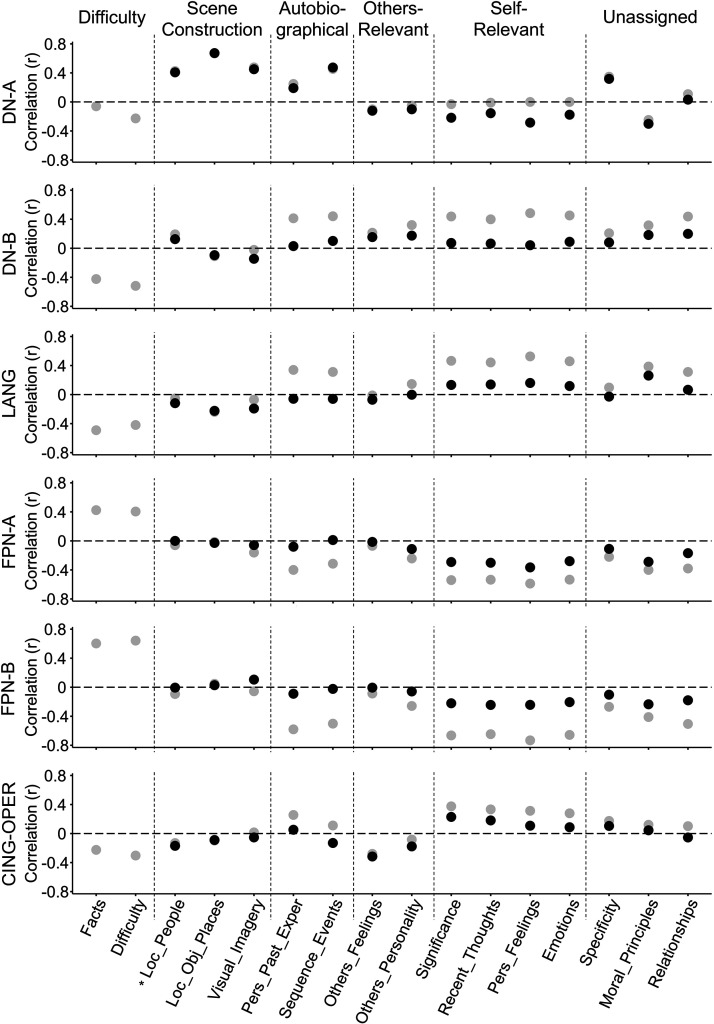
Individual strategy probes for scene construction processes show the strongest relation to DN-A response. For each individual strategy probe from the RSS, trial-level correlations to each network’s response pattern are plotted. Gray circles show correlations prior to Difficulty regression and black circles postregression. Dotted lines demarcate composites (see Fig. 4). The strategy probe for Locations of People (Loc_People, marked with a *) is grouped with the Scene Construction probes for visualization. DN-A (*top*) shows high correlations to Scene Construction probes, as well as to the Sequence_Events probe from the Autobiographical composite. DN-A is more weakly correlated to the Pers_Past_Exper probe. This pattern supports DN-A’s role in mental construction of scenes and events. Patterns across other networks show strong correlations between the FPNs and Difficulty probes (in gray, prior to regression) and a weaker but unique relation between DN-B and Others-Relevant strategies, which survives Difficulty regression. The Difficulty composite comprised Facts and Difficulty strategies, so only preregression correlations to these strategies are shown (in gray). CING-OPER, cingulo-opercular network; DN-A, default network A; DN-B, default network B; FPNs, frontoparietal control networks; LANG, language network; RSS, response strategies scale.

Plotting correlation values between DN-A response and each individual strategy probe (Fig. 15, *top*) illustrated these results, with high correlations to both Scene Construction probes (before and after regression of Difficulty), and higher correlation to Sequence_Events than Pers_Past_Exper within the Autobiographical composite. A positive correlation to Specificity (not assigned to a composite during clustering) was also revealed. Collectively, these results add to evidence for DN-A’s role in constructing mental scenes, likely including specific details of dynamic events.

## DISCUSSION

Processes linked to scene construction selectively recruited one specific distributed network, termed DN-A, that includes PHC, retrosplenial cortex, and multiple cortical association regions (see footnote 1). The interwoven but anatomically distinct DN-B showed no such response. The functional dissociation between these two juxtaposed networks was striking (Fig. 9) and suggests that DN-A is domain specialized. When functional response properties were examined broadly, across multiple distributed association networks, scene construction was associated only with DN-A response and could be further dissociated from responses tracking cognitive effort. Moreover, the relation of DN-A to scene construction held even for trials designed not to include episodic memory demands. DN-A appears to subserve scene construction processes, likely encouraged by, but not limited to, autobiographical memory tasks (see also Refs. 5, 33). We discuss the implications of these observations as well as the opportunities and limitations of our methods that leverage trial-to-trial variation in processing demands to constrain understanding of network functions.

### Trial-Level Variation in Scene Construction Robustly Tracks DN-A Response

Comparing trial-to-trial variation in Scene Construction ratings to network activity revealed a selective, strong relation to DN-A response. Prior notions that a monolithic DN makes an extremely broad processing contribution to diverse forms of mental simulation (e.g., Refs. 1, 3) are not consistent with our data. Rather, our results support the hypothesis that DN-A and DN-B contribute to distinct domains of processing, and further, that the hippocampally linked DN-A specifically subserves scene construction, including as used not only during episodic remembering and imagining future scenarios but also during atemporal imagination and for scenarios that are not necessarily relevant to oneself (see Refs. 5, 23, 33). The selectivity for processes associated with scene construction and not episodic retrieval is important to refining functional understanding.

DN-A is strongly recruited, on average, for trials featuring remembering and constructing future scenarios when these trials are contrasted to control trials, such as those involving semantic or nonpersonal reference (26; see also Refs. 3, 76). But such complex task trials rely on multiple distinct component processes (for relevant discussion, see Ref. 33). While mental scene construction presumably always uses some form of internal process as the scenes are imagined (not experienced), a clear finding is that DN-A activity is not specifically linked to whether a trial demands reliance on episodic memory, that is, retrieval from one’s own personal past. Within the entire trial set, the relation between DN-A and reported utilization of personal past experiences was weak, and when restricted to control trials, negative. However, trials, including controls, that rated low on utilizing past personal experience activated DN-A to the degree they rated high on Scene Construction. Thus, the high average response of DN-A across trials involving episodic remembering and imagining future scenarios appears driven by covariation with the more basic component process of scene construction. To the degree a trial encouraged participants to construct a mental scene with vivid imagery and awareness about spatial locations of objects or places, the response in DN-A increased. Providing further support that scene construction is the core process driving DN-A activity, contrast maps made only from control trials, using ratings on Scene Construction probes, overlapped with DN-A estimates within individuals (Fig. 13).

A nuance to interpreting our data arose in the more detailed analysis of behavioral strategies once Difficulty was regressed. Although Autobiographical ratings also tracked DN-A response, a strategy probe for envisioning event sequences largely accounted for this relation. The probe directly measuring reliance on personal past experiences more weakly related to DN-A activity. Across all probes, those for visualizing scene and event details, even including the specificity of such details, related most strongly to DN-A. This strategy pattern supported DN-A’s role in internally constructing scenes, including dynamically unfolding event sequences, even when minimally reliant on the personal past (see also Ref. 33).

The observed relations between DN-A and scene-relevant probes were not only strong but also selective. Comparing functional relations across multiple distributed association networks illustrated that only DN-A was recruited for Scene Construction. Interwoven DN-B showed almost no relation to scene-relevant strategies, nor did any of four other juxtaposed networks. And although multiple nearby networks tracked Difficulty (a composite measuring cognitive effort), DN-A showed no relation to Difficulty scores, and Difficulty-related networks did not track Scene Construction. DN-A can thus be functionally dissociated from parallel, and even interdigitated, networks within association cortex through a unique role in scene construction.

These results build evidence in support of domain specialization for individually defined, distributed network DN-A. Specifically, our evidence suggests DN-A contributes to a previously hypothesized “construction system” (33), supporting processes for mentally creating coherent scenes. These findings converge with work linking medial temporal lobe DN regions to constructing mental scenes (23; see also Refs. 77, 78) and to vivid visual imagery of events (e.g., Refs. 13, 14). And our work aligns with prior studies linking regions of DN-A to category-specific reasoning about places, including in posteromedial cortex (30–32) and in more distributed regions (21). Prior work on episodic memory has also argued for a “primacy of spatial information” during retrieval; our results could align with the hypothesis that a network of regions supports scene content that might “scaffold” memory or imagination of events (see Ref. 79; see also Ref. 80).

### Method Intuitions: Behavioral Ratings Capture Stable Properties of Trial-to-Trial Variation

In addition to network insights, the present work provided evidence that behavioral strategy ratings can tap into stable trial-level properties. If behavioral ratings varied by respondent group or by individual answers to trial questions, they would not inform independent neuroimaging data. But across two behavioral experiments, ratings of strategy probes showed striking stability. Even for individual trials, patterns were highly reliable, akin to trial-level fingerprints, and interexperiment reliability was high for nearly every strategy probe (Figs. 1 and 2). What is more, correlated strategies formed replicable clusters, suggesting that, for trials like ours (requiring consideration of different scenarios), participants have insight into how they choose a response (Figs. 3 and 4). Self-reported strategy ratings can thus capture stable trial properties, informative to independent neuroimaging results from the same trial set (see also Ref. 23).

Here, neuroimaging data were also reliable (see footnote 5) with activity levels accounting for behavioral variation. Specifically, trial-to-trial behavioral strategy ratings correlated with fMRI response activity within the individually identified networks. At first glance, our correlation results are surprising. The scatterplot showing Scene Construction scores plotted against DN-A response, for example, reveals a remarkably clean relation (Fig. 9), reminiscent of the kind of artifactual relations that emerge when there is circularity in region definition, colloquially known as double dipping (67, 81). The network regions analyzed here were defined a priori without any bias to elicit well-behaved relations. The well-behaved plots likely emerge because of the stability of the estimates. Each dot in the plots depicted in Figs. 9 and 11, for example, represents not a single person but two robust estimates, two means of reliable datasets, for a single trial. Each dot, therefore, includes a lot of data, from multiple groups of participants, stabilizing the estimate.

### Limitations and Considerations for Future Work

Despite the strengths of this approach, our datasets were limited by the trials and strategy probes we used. We asked online participants to rate strategy probes for multiple questions in a row, so we restricted the total number of probes. Additional ratings could target such dimensions as perspective, temporal orientation, emotional valence, or certainty (e.g., Refs. 47, 48). Our task could also be expanded to include additional trial questions. Trials were not explicitly designed to target social reasoning or effort, for example, which impacted our ability to probe relevant processes.

Varying levels of cognitive effort across trials appear particularly crucial to dissecting network functions. In the present work, a confounding effect of Difficulty impacted the Autobiographical and Self-Relevant composites. As discussed elsewhere, failure to control for difficulty can lead to spurious interpretations of differences between other trial features (e.g., see Ref. 82). Our task was designed to target other dimensions but nonetheless varied in difficulty, and our findings leave open questions about potential contributions of individually defined networks to complex affective, narrative, and other processes that could relate to Self-Relevant probes when difficulty is better controlled. We are cautious, for example, not to overinterpret our results in relation to the role of DN-B as our trials and probes might not sufficiently explore the relevant processes. Expanding our trial and strategy set to further examine DN-B is a goal of future work.

Considering the relevance of group-level functional insights to individuals is another important future direction. In prior analyses of these highly sampled neuroimaging participants (26), averaging across many trials allowed for interpreting results within each person. Here, to stabilize an estimate for each trial, we averaged across subjects and compared with patterns from groups of online participants. Data from a single individual could be expected to deviate from the group structure; however, Fig. 13 shows examples of individual neuroimaging data that align with group patterns, suggesting the relevance of these group-level results.

The described composite-network links also raise questions that could not be answered with the current data given the limited amount of task variance in our study. For example, though we largely focused here on the strongest network-composite relation relevant to Difficulty (i.e., FPN-B), both frontoparietal networks (FPN-A and FPN-B) showed positive correlations to the Difficulty composite score. A question for future work concerns the precise roles of these networks. Distinct contributions to aspects of control and to network coordination have been proposed (see Refs. 75, 83–85, see also Ref. 86) and yet-unappreciated roles are also a possibility (e.g., even beyond the domain of cognitive control).

In addition, the observed dissociation between DN-A and FPN-B supports a hypothesis that tightly juxtaposed association networks can distinctly subserve more domain-specialized (DN-A) or domain-general (FPN-B) processes (see also Refs. 22, 70). Whether these patterns hold across all distributed association zones is an open question. Contrast maps produced from strategy ratings showed network overlap not only in specific regions but also across distributed cortical zones for both DN-A (in relation to Scene Construction, even for control trials) and FPN-B (in relation to Difficulty; Fig. 13). These maps, along with findings of task differentiation in multiple network zones (26; see also Ref. 21), lead us to predict that functional distinctions span the cortex. We aim to test this hypothesis more directly in future work.

Finally, although DN-A has been linked to the hippocampal formation (25) and the hippocampus has long been shown to play a role in representing space (e.g., Ref. 87) and scene construction (e.g., Ref. 5; see also Ref. 88), the present analyses were limited to cortical network regions. Further examination of network connectivity to (and function of) the hippocampus itself could further clarify DN-A's functional role.

### Conclusions

Processes linked to scene construction selectively recruited DN-A. Even when examining trials explicitly designed not to rely on personal past experiences, neuroimaging contrasts created from extreme Scene Construction ratings revealed a preferential response in DN-A. Scene Construction did not recruit interwoven DN-B or four other networks, and DN-A response did not track Difficulty. These results suggest that parallel distributed networks in association cortex are functionally distinct, with DN-A making a domain-specialized processing contribution that can be robustly functionally dissociated from multiple other association networks.

## GRANTS

This work was supported by Kent and Liz Dauten, NIH grants MH124004 P50MH106435, Shared Instrumentation Grant S10OD020039. For L.M.D., this work was also supported by the National Science Foundation Graduate Research Fellowship Program under Grant No. DGE1745303, The Pershing Square Fund for Research on the Foundations of Human Behavior, and the Sigma Xi Society (GIAR G20201001117410844).

## DISCLAIMERS

Any opinions, findings, and conclusions or recommendations expressed in this material are those of the authors and do not necessarily reflect the views of the National Science Foundation.

## DISCLOSURES

No conflicts of interest, financial or otherwise, are declared by the authors.

## AUTHOR CONTRIBUTIONS

L.M.D., O.I.A., and R.L.B. conceived and designed research; L.M.D. and O.I.A. performed experiments; L.M.D., O.I.A., and R.L.B. analyzed data; L.M.D., O.I.A., and R.L.B. interpreted results of experiments; L.M.D. and R.L.B. prepared figures; L.M.D. and R.L.B. drafted manuscript; L.M.D., O.I.A., and R.L.B. edited and revised manuscript; L.M.D., O.I.A., and R.L.B. approved final version of manuscript.

## Footnotes

1 The labels DN-A and DN-B are chosen because of the literature’s description of the broader network as the default network or default mode network. DN-A and DN-B are not subnetworks of the default network. Rather, by our estimates, they are two parallel networks that have been historically mischaracterized as a composite monolithic network because of spatial blurring in group-averaged data (including in our own work; e.g., Ref. 2). Thus, the labels DN-A and DN-B are labels of convention to relate our present description of these parallel distributed networks to the historical description of the default network. The network labels should not be taken to imply strong assumptions about the functional domains of these networks (see also Refs. 9, 22 for discussion of hypotheses about potentially shared functional properties and developmental origins).

2 Composite scores were sums to mirror methods in Andrews-Hanna et al. (23), but averages could also have been used. Sum and average scores, for each composite, are necessarily perfectly correlated.

3 As described in DiNicola et al. (26), we regressed whole brain signal to remove a potential source of signal instability. Of note, we repeated the current analyses on data from which whole brain signal had not been regressed, with comparable results across networks. The greatest difference was observed in weakening the already tenuous relationship between Others-Relevant scores and DN-B recruitment, which we are careful not to overinterpret.

4 In prior work (e.g., Ref 52), we referred to this network as a candidate salience network (SAL). However, the caudal opercular region of this network, as well as insular and medial frontal regions, better align with descriptions of the cingulo-opercular network (e.g., Ref. 62) than of the salience network (e.g., Ref. 62; see also Ref. 63). We update our terminology here.

5 Split-half reliability for all composite scores was greater than 0.90 and for all networks was greater than 0.70, except for the CING-OPER network (0.52).

6 When restricting the Scene Construction map to control trials, a subset of individuals (including S1 and S7, shown) exhibited some overlap with FPN-B, as well. The subset of control trials with higher Scene Construction scores were rated as more difficult, on average, than the subset with lower Scene Construction scores (difference = 2.00). Greater (and more selective) correlation to DN-A response was observed when replacing the top 10 control with the top 10 target trials in the contrast (not shown); these target trials were rated as less difficult even than the control trials with lower Scene Construction scores (difference = −0.65).

7 For one individual (S4), the Difficulty contrast map showed more overlap with an estimate of FPN-A. As described previously, this individual had a more ambiguous *k*-means output (S11 in Ref. 26), and this result likely reflects uncertainty in network assignment, rather than true network differences, in this subject. This result raises the importance of exploring network responses in additional individuals.

8 Within this model, the two Scene Construction probes were highly intercorrelated (and VIF > 3). A similar model, with these probes combined, produced comparable results. Most DN-A variance was captured by Scene Construction (*R*^2^_Scene Construction_ = 0.25, *P* < 0.001; *R*^2^_Full Model_ = 0.40), followed by Sequence_Events (*R*^2^ = 0.12, *P* < 0.001), and almost none by Pers_Past_Exper (*R*^2^ = 0.02, *P* < 0.05).

## References

[1] Buckner RL, Carroll DC. Self-projection and the brain. Trends Cogn Sci 11: 49–57, 2007. doi:10.1016/j.tics.2006.11.004. 17188554

[2] Buckner RL, Andrews-Hanna JR, Schacter DL. The brain’s default network: anatomy, function, and relevance to disease. Ann N Y Acad Sci 1124: 1–38, 2008. doi:10.1196/annals.1440.011. 18400922

[3] Spreng RN, Mar RA, Kim ASN. The common neural basis of autobiographical memory, prospection, navigation, theory of mind, and the default mode: a quantitative meta-analysis. J Cogn Neurosci 21: 489–510, 2009. doi:10.1162/jocn.2008.21029. 18510452

[4] Gusnard DA, Raichle ME. Searching for a baseline: functional imaging and the resting human brain. Nat Rev Neurosci 2: 685–694, 2001. doi:10.1038/35094500. 11584306

[5] Hassabis D, Maguire EA. Deconstructing episodic memory with construction. Trends Cogn Sci 11: 299–306, 2007. doi:10.1016/j.tics.2007.05.001. 17548229

[6] Schacter DL, Addis DR, Buckner RL. Remembering the past to imagine the future: the prospective brain. Nat Rev Neurosci 8: 657–661, 2007. doi:10.1038/nrn2213. 17700624

[7] Binder JR, Desai RH, Graves WW, Conant LL. Where is the semantic system? A critical review and meta-analysis of 120 functional neuroimaging studies. Cereb Cortex 19: 2767–2796, 2009. doi:10.1093/cercor/bhp055. 19329570 PMC2774390

[8] Margulies DS, Ghosh SS, Goulas A, Falkiewicz M, Huntenburg JM, Langs G, Bezgin G, Eickhoff SB, Castellanos FX, Petrides M, Jefferies E, Smallwood J. Situating the default-mode network along a principal gradient of macroscale cortical organization. Proc Natl Acad Sci USA 113: 12574–12579, 2016. doi:10.1073/pnas.1608282113. 27791099 PMC5098630

[9] Buckner RL, DiNicola LM. The brain’s default network: updated anatomy, physiology and evolving insights. Nat Rev Neurosci 20: 593–608, 2019. doi:10.1038/s41583-019-0212-7. 31492945

[10] Murphy C, Jefferies E, Rueschemeyer S-A, Sormaz M, Wang H-T, Margulies DS, Smallwood J. Distant from input: evidence of regions within the default mode network supporting perceptually-decoupled and conceptually-guided cognition. Neuroimage 171: 393–401, 2018. doi:10.1016/j.neuroimage.2018.01.017. 29339310 PMC5883322

[11] Sormaz M, Murphy C, Wang H-T, Hymers M, Karapanagiotidis T, Poerio G, Margulies DS, Jefferies E, Smallwood J. Default mode network can support the level of detail in experience during active task states. Proc Natl Acad Sci USA 115: 9318–9323, 2018 [Erratum in *Proc Natl Acad Sci USA* 115: E11198, 2018]. doi:10.1073/pnas.1721259115. 30150393 PMC6140531

[12] Beaty RE, Chen Q, Christensen AP, Kenett YN, Silvia PJ, Benedek M, Schacter DL. Default network contributions to episodic and semantic processing during divergent creative thinking: a representational similarity analysis. Neuroimage 209: 116499, 2020. doi:10.1016/j.neuroimage.2019.116499. 31887423 PMC7056499

[13] Lee S, Parthasarathi T, Kable JW. The ventral and dorsal default mode networks are dissociably modulated by the vividness sand valence of imagined events. J Neurosci 41: 5243–5250, 2021. doi:10.1523/JNEUROSCI.1273-20.2021. 34001631 PMC8211541

[14] Wen T, Mitchell DJ, Duncan J. The functional convergence and heterogeneity of social, episodic, and self-referential thought in the default mode network. Cereb Cortex 30: 5915–5929, 2020. doi:10.1093/cercor/bhaa166. 32572493 PMC7116230

[15] Yeshurun Y, Nguyen M, Hasson U. The default mode network: where the idiosyncratic self meets the shared social world. Nat Rev Neurosci 22: 181–192, 2021. doi:10.1038/s41583-020-00420-w. 33483717 PMC7959111

[16] Mancuso L, Cavuoti-Cabanillas S, Liloia D, Manuello J, Buzi G, Cauda F, Costa T. Tasks activating the default mode network map multiple functional systems. Brain Struct Funct 227: 1711–1734, 2022. doi:10.1007/s00429-022-02467-0. 35179638 PMC9098625

[17] Steinmetz H, Seitz RJ. Functional anatomy of language processing: neuroimaging and the problem of individual variability. Neuropsychologia 29: 1149–1161, 1991. doi:10.1016/0028-3932(91)90030-c. 1791929

[18] Fedorenko E, Hsieh P-J, Nieto-Castañón A, Whitfield-Gabrieli S, Kanwisher N. New method for fMRI investigations of language: defining ROIs functionally in individual subjects. J Neurophysiol 104: 1177–1194, 2010. doi:10.1152/jn.00032.2010. 20410363 PMC2934923

[19] Laumann TO, Gordon EM, Adeyemo B, Snyder AZ, Joo SJ, Chen M-Y, Gilmore AW, McDermott KB, Nelson SM, Dosenbach NUF, Schlaggar BL, Mumford JA, Poldrack RA, Petersen SE. Functional system and areal organization of a highly sampled individual human brain. Neuron 87: 657–670, 2015. doi:10.1016/j.neuron.2015.06.037. 26212711 PMC4642864

[20] Braga RM, Buckner RL. Parallel interdigitated distributed networks within the individual estimated by intrinsic functional connectivity. Neuron 95: 457–471.e5, 2017. doi:10.1016/j.neuron.2017.06.038. 28728026 PMC5519493

[21] Deen B, Friewald WA. Parallel systems for social and spatial reasoning within the cortical apex. *bioRxiv*, 2022. doi:10.1101/2021.09.23.461550. PMC12595413 41166425

[22] DiNicola LM, Buckner RL. Precision estimates of parallel distributed association networks: evidence for domain specialization and implications for evolution and development. Curr Opin Behav Sci 40: 120–129, 2021. doi:10.1016/j.cobeha.2021.03.029. 34263017 PMC8274557

[23] Andrews-Hanna JR, Reidler JS, Sepulcre J, Poulin R, Buckner RL. Functional-anatomic fractionation of the brain’s default network. Neuron 65: 550–562, 2010. doi:10.1016/j.neuron.2010.02.005. 20188659 PMC2848443

[24] Yeo BTT, Krienen FM, Sepulcre J, Sabuncu MR, Lashkari D, Hollinshead M, Roffman JL, Smoller JW, Zöllei L, Polimeni JR, Fischl B, Liu H, Buckner RL. The organization of the human cerebral cortex estimated by intrinsic functional connectivity. J Neurophysiol 106: 1125–1165, 2011. doi:10.1152/jn.00338.2011. 21653723 PMC3174820

[25] Braga RM, Van Dijk KRA, Polimeni JR, Eldaief MC, Buckner RL. Parallel distributed networks resolved at high resolution reveal close juxtaposition of distinct regions. J Neurophysiol 121: 1513–1534, 2019. doi:10.1152/jn.00808.2018. 30785825 PMC6485740

[26] DiNicola LM, Braga RM, Buckner RL. Parallel distributed networks dissociate episodic and social functions within the individual. J Neurophysiol 123: 1144–1179, 2020. [Erratum in *J Neurophysiol* 124: 307, 2020]. doi:10.1152/jn.00529.2019. 32049593 PMC7099479

[27] Rosenbaum RS, Stuss DT, Levine B, Tulving E. Theory of mind is independent of episodic memory. Science 318: 1257, 2007. doi:10.1126/science.1148763. 18033875

[28] Andrews-Hanna JR, Saxe R, Yarkoni T. Contributions of episodic retrieval and mentalizing to autobiographical thought: Evidence from functional neuroimaging, resting-state connectivity, and fMRI meta-analyses. Neuroimage 91: 324–335, 2014. doi:10.1016/j.neuroimage.2014.01.032. 24486981 PMC4001766

[29] Kurczek J, Wechsler E, Ahuja S, Jensen U, Cohen NJ, Tranel D, Duff M. Differential contributions of hippocampus and medial prefrontal cortex to self-projection and self-referential processing. Neuropsychologia 73: 116–126, 2015. doi:10.1016/j.neuropsychologia.2015.05.002. 25959213 PMC4671497

[30] Peer M, Salomon R, Goldberg I, Blanke O, Arzy S. Brain system for mental orientation in space, time, and person. Proc Natl Acad Sci USA 112: 11072–11077, 2015. doi:10.1073/pnas.1504242112. 26283353 PMC4568229

[31] Silson EH, Steel A, Kidder A, Gilmore AW, Baker CI. Distinct subdivisions of human medial parietal cortex support recollection of people and places. eLife 8: e47391, 2019. doi:10.7554/eLife.47391. 31305238 PMC6667275

[32] Woolnough O, Rollo PS, Forseth KJ, Kadipasaoglu CM, Ekstrom AD, Tandon N. Category selectivity for face and scene recognition in human medial parietal cortex. Curr Biol 30: 2707–2715.e3, 2020. doi:10.1016/j.cub.2020.05.018. 32502406 PMC10322184

[33] Hassabis D, Maguire EA. The construction system of the brain. Philos Trans R Soc Lond B Biol Sci 364: 1263–1271, 2009. doi:10.1098/rstb.2008.0296. 19528007 PMC2666702

[34] Schacter DL, Addis DR, Hassabis D, Martin VC, Spreng RN, Szpunar KK. The future of memory: remembering, imagining, and the brain. Neuron 76: 677–694, 2012. doi:10.1016/j.neuron.2012.11.001. 23177955 PMC3815616

[35] Tulving E. Elements of Episodic Memory. Oxford, UK: Oxford University Press, 1983.

[36] Kopelman MD. Frontal dysfunction and memory deficits in the alcoholic Korsakoff Syndrome and Alzheimer-type dementia. Brain 114: 117–137, 1991. 1998878

[37] Dobbins IG, Foley H, Schacter DL, Wagner AD. Executive control during episodic retrieval: multiple prefrontal processes subserve source memory. Neuron 35: 989–996, 2002. doi:10.1016/s0896-6273(02)00858-9. 12372291

[38] Bunge SA, Burrows B, Wagner AD. Prefrontal and hippocampal contributions to visual associative recognition: interactions between cognitive control and episodic retrieval. Brain Cogn 56: 141–152, 2004. doi:10.1016/j.bandc.2003.08.001. 15518931

[39] Vatansever D, Smallwood J, Jefferies E. Varying demands for cognitive control reveals shared neural processes supporting semantic and episodic memory retrieval. Nat Commun 12: 2134, 2021. doi:10.1038/s41467-021-22443-2. 33837220 PMC8035200

[40] Moscovitch M. Memory and working-with-memory: a component process model based on modules and central systems. J Cog Neurosci 4: 257–267, 1992. doi:10.1162/jocn.1992.4.3.257. 23964882

[41] Badre D, Wagner AD. Left ventrolateral prefrontal cortex and the cognitive control of memory. Neuropsychologia 45: 2883–2901, 2007. doi:10.1016/j.neuropsychologia.2007.06.015. 17675110

[42] Kirchhoff BA, Buckner RL. Functional-anatomic correlates of individual differences in memory. Neuron 51: 263–274, 2006. doi:10.1016/j.neuron.2006.06.006. 16846860

[43] Skerry AE, Saxe R. Neural representations of emotion are organized around abstract event features. Curr Biol 25: 1945–1954, 2015. doi:10.1016/j.cub.2015.06.009. 26212878 PMC4824044

[44] Bruneau E, Dufour N, Saxe R. How we know it hurts: Item analysis of written narratives reveals distinct neural responses to others’ physical pain and emotional suffering. PLoS One 8: e63085, 2013. doi:10.1371/journal.pone.0063085. 23638181 PMC3637309

[45] Litman L, Robinson J, Abberbock T. TurkPrime.com: a versatile crowdsourcing data acquisition platform for the behavioral sciences. Behav Res Methods 49: 433–442, 2017. doi:10.3758/s13428-016-0727-z. 27071389 PMC5405057

[46] Sutin AR, Robins RW. Phenomenology of autobiographical memories: the memory experiences questionnaire. Memory 15: 390–411, 2007. doi:10.1080/09658210701256654. 17469019

[47] Stawarczyk D, Cassol H, D’Argembeau A. Phenomenology of future-oriented mind-wandering episodes. Front Psychol 4: 425, 2013. doi:10.3389/fpsyg.2013.00425. 23882236 PMC3712143

[48] Andrews-Hanna JR, Kaiser RH, Turner AEJ, Reineberg AE, Godinez D, Dimidjian S, Banich MT. A penny for your thoughts: dimensions of self-generated thought content and relationships with individual differences in emotional wellbeing. Front Psychol 4: 900, 2013. doi:10.3389/fpsyg.2013.00900. 24376427 PMC3843223

[49] Poerio GL, Sormaz M, Wang H-T, Margulies D, Jefferies E, Smallwood J. The role of the default mode network in component processes underlying the wandering mind. Soc Cogn Affect Neurosci 12: 1047–1062, 2017. doi:10.1093/scan/nsx041. 28402561 PMC5490683

[50] Johnson MK, Foley MA, Suengas AG, Raye CL. Phenomenal characteristics of memories for perceived and imagined autobiographical events. J Exp Psychol Gen 117: 371–376, 1988. 2974863

[51] Buchanan EM, Scofield JE. Methods to detect low quality data and its implication for psychological research. Behav Res Methods 50: 2586–2596, 2018. doi:10.3758/s13428-018-1035-6. 29542063

[52] Braga RM, DiNicola LM, Becker HC, Buckner RL. Situating the left-lateralized language network in the broader organization of multiple specialized large-scale distributed networks. J Neurophysiol 124: 1415–1448, 2020. doi:10.1152/jn.00753.2019. 32965153 PMC8356783

[53] van der Kouwe AJW, Benner T, Salat DH, Fischl B. Brain morphometry with multiecho MPRAGE. Neuroimage 40: 559–569, 2008. doi:10.1016/j.neuroimage.2007.12.025. 18242102 PMC2408694

[54] Feinberg DA, Moeller S, Smith SM, Auerbach E, Ramanna S, Gunther M, Glasser MF, Miller KL, Ugurbil K, Yacoub E. Multiplexed echo planar imaging for sub-second whole brain FMRI and fast diffusion imaging. PLoS One 5: e15710, 2010 [Erratum in *PLoS One* 6: 10.1371/annotation/d9496d01-8c5d-4d24-8287-94449ada5064]. doi:10.1371/annotation/d9496d01-8c5d-4d24-8287-94449ada5064. 21187930 PMC3004955

[55] Moeller S, Yacoub E, Olman CA, Auerbach E, Strupp J, Harel N, Uğurbil K. Multiband multislice GE-EPI at 7 tesla, with 16-fold acceleration using partial parallel imaging with application to high spatial and temporal whole-brain fMRI. Magn Reson Med 63: 1144–1153, 2010. doi:10.1002/mrm.22361. 20432285 PMC2906244

[56] Setsompop K, Gagoski BA, Polimeni JR, Witzel T, Wedeen VJ, Wald LL. Blipped-controlled aliasing in parallel imaging for simultaneous multislice echo planar imaging with reduced g-factor penalty. Magn Reson Med 67: 1210–1224, 2012. doi:10.1002/mrm.23097. 21858868 PMC3323676

[57] Xu J, Moeller S, Auerbach EJ, Strupp J, Smith SM, Feinberg DA, Yacoub E, Uğurbil K. Evaluation of slice accelerations using multiband echo planar imaging at 3T. Neuroimage 83: 991–1001, 2013. doi:10.1016/j.neuroimage.2013.07.055. 23899722 PMC3815955

[58] Cox RW. AFNI: software for analysis and visualization of functional magnetic resonance neuroimages. Comput Biomed Res 29: 162–173, 1996. doi:10.1006/cbmr.1996.0014. 8812068

[59] Cox RW. AFNI: What a long strange trip it’s been. Neuroimage 62: 743–747, 2012. doi:10.1016/j.neuroimage.2011.08.056. 21889996 PMC3246532

[60] Fischl B, Sereno MI, Dale AM. Cortical surface-based analysis: II: inflation, flattening, and a surface-based coordinate system. Neuroimage 9: 195–207, 1999. doi:10.1006/nimg.1998.0396. 9931269

[61] Holmes AJ, Hollinshead MO, O’Keefe TM, Petrov VI, Fariello GR, Wald LL, Fischl B, Rosen BR, Mair RW, Roffman JL, Smoller JW, Buckner RL. Brain Genomics Superstruct Project initial data release with structural, functional, and behavioral measures. Sci Data 2: 150031, 2015. doi:10.1038/sdata.2015.31. 26175908 PMC4493828

[62] Seeley WW, Menon V, Schatzberg AF, Keller J, Glover GH, Kenna H, Reiss AL, Greicius MD. Dissociable intrinsic connectivity networks for salience processing and executive control. J Neurosci 27: 2349–2356, 2007. doi:10.1523/JNEUROSCI.5587-06.2007. 17329432 PMC2680293

[63] Seeley WW. The salience network: a neural system for perceiving and responding to homeostatic demands. J Neurosci 39: 9878–9882, 2019. doi:10.1523/jneurosci.1138-17.2019. 31676604 PMC6978945

[64] Hassabis D, Spreng RN, Rusu AA, Robbins CA, Mar RA, Schacter DL. Imagine all the people: how the brain creates and uses personality models to predict behavior. Cereb Cortex 24: 1979–1987, 2014. doi:10.1093/cercor/bht042. 23463340 PMC4089378

[65] Groemping U. Relative importance for linear regression in R: The package relaimpo. J Stat Soft 17: 1–27, 2006. doi:10.18637/jss.v017.i01.

[66] Konkle T, Brady TF, Alvarez GA, Oliva A. Conceptual distinctiveness supports detailed visual long-term memory for real-world objects. J Exp Psychol Gen 139: 558–578, 2010. doi:10.1037/a0019165. 20677899 PMC3398125

[67] Vul E, Harris C, Winkielman P, Pashler H. Puzzlingly high correlations in fMRI studies of emotion, personality, and social cognition. Perspect Psychol Sci 4: 274–290, 2009. doi:10.1111/j.1745-6924.2009.01125.x. 26158964

[68] Marcus DS, Harwell J, Olsen T, Hodge M, Glasser MF, Prior F, Jenkinson M, Laumann T, Curtiss SW, Van Essen DC. Informatics and data mining tools and strategies for the Human Connectome Project. Front Neuroinform 5: 4, 2011. doi:10.3389/fninf.2011.00004. 21743807 PMC3127103

[69] Johnston R, Jones K, Manley D. Confounding and collinearity in regression analysis: a cautionary tale and an alternative procedure, illustrated by studies of British voting behaviour. Qual Quant 52: 1957–1976, 2018. doi:10.1007/s11135-017-0584-6. 29937587 PMC5993839

[70] Fedorenko E, Duncan J, Kanwisher N. Language-selective and domain-general regions lie side by side within Broca’s area. Curr Biol 22: 2059–2062, 2012. doi:10.1016/j.cub.2012.09.011. 23063434 PMC3494832

[71] Lieberman MD. Social cognitive neuroscience: a review of core processes. Annu Rev Psychol 58: 259–289, 2007. doi:10.1146/annurev.psych.58.110405.085654. 17002553

[72] Koster-Hale J, Saxe R. Functional neuroimaging of theory of mind. In: Understanding Other Minds: Perspectives from Developmental Social Neuroscience (3rd ed.), edited by Baron-Cohen S, Lombardo M, Tager-Flusberg H. Oxford, UK: Oxford University Press, 2013.

[73] Schurz M, Radua J, Tholen MG, Maliske L, Margulies DS, Mars RB, Sallet J, Kanske P. Toward a hierarchical model of social cognition: a neuroimaging meta-analysis and integrative review of empathy and theory of mind. Psychol Bull 147: 293–327, 2021. doi:10.1037/bul0000303. 33151703

[74] Miller EK, Cohen JD. An integrative theory of prefrontal cortex function. Annu Rev Neurosci 24: 167–202, 2001. doi:10.1146/annurev.neuro.24.1.167. 11283309

[75] Badre D, Nee DE. Frontal cortex and the hierarchical control of behavior. Trends Cogn Sci 22: 170–188, 2018. doi:10.1016/j.tics.2017.11.005. 29229206 PMC5841250

[76] Addis DR, Wong AT, Schacter DL. Remembering the past and imagining the future: common and distinct neural substrates during event construction and elaboration. Neuropsychologia 45: 1363–1377, 2007. doi:10.1016/j.neuropsychologia.2006.10.016. 17126370 PMC1894691

[77] Axelrod V, Rees G, Bar M. The default network and the combination of cognitive processes that mediate self-generated thought. Nat Hum Behav 1: 896–910, 2017. doi:10.1038/s41562-017-0244-9. 30035236 PMC6054300

[78] Palombo DJ, Hayes SM, Peterson KM, Keane MM, Verfaellie M. Medial temporal lobe contributions to episodic future thinking: scene construction or future projection? Cereb Cortex 28: 447–458, 2018. doi:10.1093/cercor/bhw381. 27913433 PMC5965081

[79] Robin J. Spatial scaffold effects in event memory and imagination. Wiley Interdiscip Rev Cogn Sci 9: e1462, 2018. doi:10.1002/wcs.1462. 29485243

[80] Whittington JCR, Muller TH, Mark S, Chen G, Barry C, Burgess N, Behrens TEJ. The Tolman-Eichenbaum machine: unifying space and relational memory through generalization in the hippocampal formation. Cell 183: 1249–1263.e23, 2020. doi:10.1016/j.cell.2020.10.024. 33181068 PMC7707106

[81] Kriegeskorte N, Simmons WK, Bellgowan PSF, Baker CI. Circular analysis in systems neuroscience: the dangers of double dipping. Nat Neurosci 12: 535–540, 2009. doi:10.1038/nn.2303. 19396166 PMC2841687

[82] Caramazza A, Shelton JR. Domain-specific knowledge systems in the brain: the animate-inanimate distinction. J Cogn Neurosci 10: 1–34, 1998. doi:10.1162/089892998563752. 9526080

[83] Dixon ML, De La Vega A, Mills C, Andrews-Hanna J, Spreng RN, Cole MW, Christoff K. Heterogeneity within the frontoparietal control network and its relationship to the default and dorsal attention networks. Proc Nat Acad Sci USA 115: E1598–E1607, 2018 [Erratum in *Proc Nat Acad Sci USA* 115: E3068, 2018]. doi:10.1073/pnas.1715766115. 29382744 PMC5816169

[84] Murphy AC, Bertolero MA, Papadopoulos L, Lydon-Staley DM, Bassett DS. Multimodal network dynamics underpinning working memory. Nat Commun 11: 3035, 2020. doi:10.1038/s41467-020-15541-0. 32541774 PMC7295998

[85] Nee DE. Integrative frontal-parietal dynamics supporting cognitive control. eLife 10: e57244, 2021. doi:10.7554/eLife.57244. 33650966 PMC7963482

[86] Marek S, Dosenbach NUF. The frontoparietal network: function, electrophysiology, and importance of individual precision mapping. Dialogues Clin Neurosci 20: 133–140, 2018. doi:10.31887/dcns.2018.20.2/smarek. 30250390 PMC6136121

[87] O’Keefe J, Dostrovsky J. The hippocampus as a spatial map. Preliminary evidence from unit activity in the freely-moving rat. Brain Res 34: 171–175, 1971. doi:10.1016/0006-8993(71)90358-1. 5124915

[88] Maguire EA, Intraub H, Mullally SL. Scenes, spaces, and memory traces: what does the hippocampus do? Neuroscientist 22: 432–439, 2016. doi:10.1177/1073858415600389. 26276163 PMC5021215

